# Increasing extracellular H_2_O_2_ produces a bi-phasic response in intracellular H_2_O_2_, with peroxiredoxin hyperoxidation only triggered once the cellular H_2_O_2_-buffering capacity is overwhelmed

**DOI:** 10.1016/j.freeradbiomed.2016.02.035

**Published:** 2016-06

**Authors:** Lewis Elwood Tomalin, Alison Michelle Day, Zoe Elizabeth Underwood, Graham Robert Smith, Piero Dalle Pezze, Charalampos Rallis, Waseema Patel, Bryan Craig Dickinson, Jürg Bähler, Thomas Francis Brewer, Christopher Joh-Leung Chang, Daryl Pierson Shanley, Elizabeth Ann Veal

**Affiliations:** a Institute for Cell and Molecular Biosciences, Newcastle University, Framlington Place, Newcastle upon Tyne NE2 4HH, UK; b Bioinformatics Support Unit, Newcastle University, Framlington Place, Newcastle upon Tyne NE2 4HH, UK; c University College London, Department of Genetics, Evolution & Environment and Institute of Healthy Ageing, Gower Street – Darwin Building, London WC1E 6BT, UK; d Department of Chemistry, The University of Chicago, Chicago, IL 60637, USA; e Howard Hughes Medical Institute and Departments of Chemistry and Molecular and Cell Biology, University of California, Berkeley, Berkeley, CA 94720, USA

**Keywords:** Prx, peroxiredoxins, Trx, thioredoxin, Txl1, thioredoxin-like protein 1, Gpx1, glutathione peroxidase 1, AMS, 4-acetamido-4ʹ-((iodoacetyl)amino)stilbene-2,2ʹ-disulfonic acid, NEM, N-ethylmaleimide, ROS, reactive oxygen species, H_2_O_2_, hydrogen peroxide, CysP, peroxidatic cysteine, CysR, resolving cysteine, Pr-SS, protein disulfides, Pr-SH, protein thiols, DMEM, Dulbecco’s Modified Eagles Medium, EMM, Edinburgh minimal media, TCA, trichloroacetic acid, DPBS, Dulbecco’s phosphate-buffered saline, HRP, horse radish peroxidase, PF3, acetylated peroxyfluorescein 3, AIC, Akaike Information Criterion, Peroxiredoxin, Hydrogen peroxide, Thiol, Computational model, Signaling, Oxidation, Thioredoxin

## Abstract

Reactive oxygen species, such as H_2_O_2_, can damage cells but also promote fundamental processes, including growth, differentiation and migration. The mechanisms allowing cells to differentially respond to toxic or signaling H_2_O_2_ levels are poorly defined. Here we reveal that increasing external H_2_O_2_ produces a bi-phasic response in intracellular H_2_O_2_. Peroxiredoxins (Prx) are abundant peroxidases which protect against genome instability, ageing and cancer. We have developed a dynamic model simulating in vivo changes in Prx oxidation. Remarkably, we show that the thioredoxin peroxidase activity of Prx does not provide any significant protection against external rises in H_2_O_2_. Instead, our model and experimental data are consistent with low levels of extracellular H_2_O_2_ being efficiently buffered by other thioredoxin-dependent activities, including H_2_O_2_-reactive cysteines in the thiol-proteome. We show that when extracellular H_2_O_2_ levels overwhelm this buffering capacity, the consequent rise in intracellular H_2_O_2_ triggers hyperoxidation of Prx to thioredoxin-resistant, peroxidase-inactive form/s. Accordingly, Prx hyperoxidation signals that H_2_O_2_ defenses are breached, diverting thioredoxin to repair damage.

## Highlights

• Mathematical model simulating *in vivo* dynamic, H_2_O_2_-induced, changes in Prx oxidation.
• Increasing extracellular H_2_O_2_ produces a bi-phasic response in intracellular H_2_O_2_.
• Thioredoxin and the thiol-proteome can efficiently buffer low levels of H_2_O_2_.
• Prx hyperoxidation only occurs once this H_2_O_2_ buffering capacity is saturated.

## Graphical abstract

**Figure:**
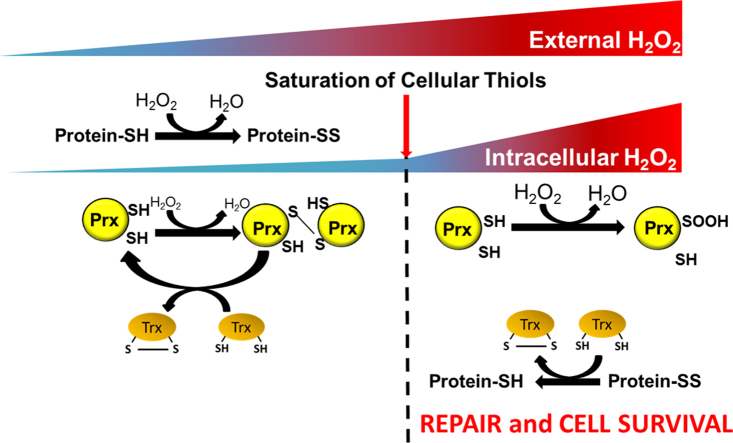

## 1 Introduction

Reactive oxygen species (ROS) generated by the partial reduction of oxygen during aerobic metabolism, immune cell attack or following exposure to radiation can cause lethal levels of cell damage. Hence, there is a strong driving force to evolve and maintain ROS-protective mechanisms. Nevertheless, altered redox homeostasis and increased oxidative cell damage are associated with the development of many common diseases, including cancer, diabetes, cardiovascular and neurodegenerative diseases. However, there is increasing evidence that low levels of ROS can also have beneficial effects; acting as signaling molecules to regulate diverse biological processes (for a review see [1]). Hence, there is considerable interest in understanding how cells optimize ROS defenses to provide adequate protection without compromising ROS-signaling functions. Here we have developed and used a mathematical model as a tool to understand what governs how cells respond to increases in hydrogen peroxide (H_2_O_2_).

**Fig. 1:**
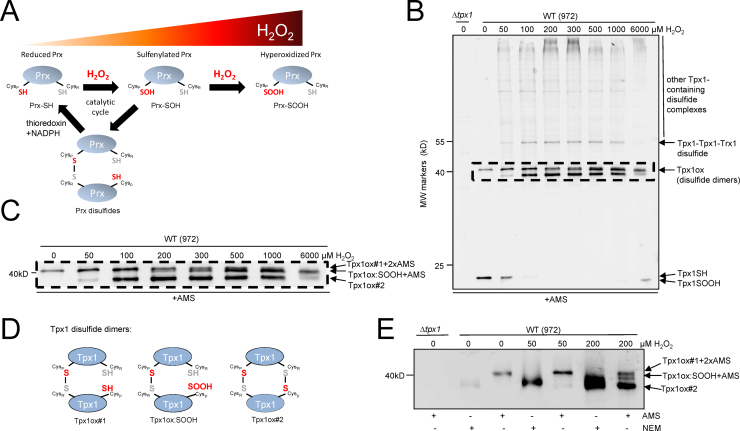
The Prx, Tpx1, undergoes oxidation to multiple redox states following exposure to different concentrations of H_2_O_2_: (A) the thioredoxin peroxidase activity of Prx, such as Tpx1, involves the reversible oxidation of catalytic cysteines but is inactivated by hyperoxidation of the peroxidatic cysteine at high concentrations of H_2_O_2_. The catalytic breakdown of H_2_O_2_ by 2-Cys peroxiredoxins (Prx) involves the reaction of the peroxidatic cysteine (Cys_P_) with H_2_O_2_. In the catalytic cycle the sulfenylated cysteine is stabilized by forming a disulfide with the resolving cysteine (Cys_R_) in a neighboring Prx molecule. These Prx disulfides are reduced by the thioredoxin system using electrons from NADPH. In eukaryotes Prx disulfide formation is slow, rendering the sulfenylated Prx, Prx-SOH, susceptible to further oxidation to a sulfinylated, ‘hyperoxidized’ form (Prx-SOOH) that cannot be reduced by thioredoxin. This hyperoxidation is favored at higher concentrations of H_2_O_2_ inactivating the thioredoxin peroxidase activity of the Prx. Western blot analysis (anti-Tpx1 antibodies) of (B) and (C) AMS-treated protein extracts from wild-type (972) and Δ*tpx1* mutant (VX00) cells treated, as indicated, for 20 s with 0–6 mM H_2_O_2_ reveals that Tpx1 undergoes oxidation to a number of redox states following exposure to H_2_O_2_. The absence of bands in ∆*tpx1* mutant (VX00) cells indicates that all the bands detected in wild-type cells represent Tpx1 or Tpx1-containing complexes. (C) A magnified image of the ~40 kDa region outlined by the dotted line in (B) shows that 3 different Tpx1-containing disulfide dimers (Tpx1ox) are detected following treatment with concentrations ≥200 µM. (D) Different Tpx1ox forms are depicted which were separated in (E) on the basis of the reduced mobility associated with modification of free cysteine thiols by AMS (0.6 kDa) compared with NEM (0.1 kDa). (E) disulfide dimers (Tpx1ox) in duplicate samples extracted from wild-type or Δ*tpx1* mutant (VX00) cells before or following treatment for 20 s with the indicated concentration of H_2_O_2_ then reacted with AMS or NEM. As in (B), only low levels of Tpx1ox (disulfides) are detected in untreated cells or cells treated with 50 µM. See also Table S1 and Fig. S1.

Peroxiredoxins (Prx) are amongst the most prevalent enzymes involved in responses to H_2_O_2_. Prx are ubiquitous and highly expressed peroxidases which utilize reversibly oxidized cysteine residues to reduce peroxides (for a review see [2]) (Fig. 1A). Intriguingly, peroxiredoxins appear to have dual roles in cancer, acting as tumor suppressors but with increased Prx expression also associated with poor prognosis tumors and increased metastasis. Prx have also been shown to promote longevity in yeast, worms, flies and mammals (for a review see [3]). There is therefore great interest in understanding how Prx influence cell responses to H_2_O_2_.

The catalytic mechanism of the typical 2-Cys Prx subfamily involves the initial reaction of an active, peroxidatic cysteine (Cys_P_) with peroxide to form a cysteine-sulfenic acid (SOH) (Fig. 1A). The sulfenylated peroxidatic cysteine then forms a disulfide with a second, resolving cysteine (Cys_R_) in an adjacent Prx molecule. In eukaryotes, these Prx disulfides are reduced by the thioredoxin system. However, the sulfenylated peroxidatic cysteines of thioredoxin-coupled Prx are highly susceptible to further ‘hyperoxidation’ to thioredoxin-resistant sulfinic-derivatives, thus inactivating their thioredoxin peroxidase activity [4] (Fig. 1A). In contrast, bacterial 2-Cys Prx, such as the *E. coli* peroxiredoxin AhpC, are much less sensitive to hyperoxidation [5]. Conserved YF and GG(L/V/I)G amino acid motifs found in all hyperoxidation-sensitive Prx are responsible for this sensitivity [5]. The evolution of these conserved amino acid motifs suggests that Prx hyperoxidation confers a selective advantage in eukaryotes. Indeed, as described below, several possible functions have been proposed for hyperoxidation of Prx.

In eukaryotes, in which Prx are sensitive to inactivation, H_2_O_2_ is generated and utilized as a signaling molecule [2]. Thus it has been proposed that the thioredoxin peroxidase activity of Prx might act as a barrier to this signaling, and that inactivation of Prx might be important to allow H_2_O_2_ to regulate target proteins [5]. Although, Prx are not hyperoxidized in response to the low levels of H_2_O_2_ generated in response to growth factors [6], oscillations in the amount of hyperoxidized Prx have been associated with circadian rhythms across a wide range of species (for a review see [7]). Moreover, oscillations in the hyperoxidation of the mouse mitochondrial Prx, Prx3, have been shown to be important for circadian oscillations in p38 activation and adrenal steroid synthesis [8]. Nevertheless, it remains unclear whether the hyperoxidation of Prx in any of these contexts serves to increase the levels of H_2_O_2_ available for signaling. Moreover, work in the fission yeast *Schizosaccharomyces pombe* has shown that, rather than acting as a barrier, the thioredoxin peroxidase activity of the single *S. pombe* peroxiredoxin, Tpx1, is actually required for the H_2_O_2_-induced activation of the AP-1-like transcription factor Pap1 [9], [10], [11]. We have shown that the role of the thioredoxin peroxidase activity of Tpx1 in H_2_O_2_-induced Pap1 activation is to competitively inhibit the reduction of the active, oxidized form of Pap1 by the thioredoxin-like protein, Txl1 [11]. Accordingly, hyperoxidation of Tpx1 to a thioredoxin-resistant form prevents the H_2_O_2_-induced activation of Pap1 by increasing the availability of reduced Txl1 [11]. Based on these studies, it has been proposed that hyperoxidation of Tpx1 to a thioredoxin-resistant form is important to increase the pool of reduced thioredoxin available to repair oxidatively damaged proteins and support the activity of other enzymes, such as the methionine sulfoxide reductase, Mxr1 [12]. Consistent with this hypothesis, hyperoxidation of Tpx1 and the *C. elegans* peroxiredoxin PRDX-2 are important for cell survival under acute stress conditions [12], [13]. Hyperoxidation of Prx has also been proposed to have other roles, for example, protecting cells against protein aggregation by promoting the ability of Prx to act as a chaperone [14]. However, it is still unclear under what conditions extensive Prx hyperoxidation occurs *in vivo* and when and where this might be important.

Although all eukaryotic Prx are inherently sensitive to hyperoxidation, other factors affect this sensitivity. For example, *in vitro* studies revealed that human cytosolic Prx1 is much more sensitive to hyperoxidation than the mitochondrial Prx, Prx3 [15]. This increased sensitivity reflects a 10-fold slower rate of Prx1 disulfide formation that increases the risk of further oxidation of the sulfenylated peroxidatic cysteine [16], [17]. In addition to the intrinsic biochemical properties of the Prx itself, the *in vivo* sensitivity of Prx to hyperoxidation is also influenced by the local environment. For example, although *in vitro* the ER-localized Prx, Prx4, has a similar sensitivity to hyperoxidation to Prx1, a negligible proportion of Prx4 becomes hyperoxidized to its sulfinic form *in vivo*. This is due to the low abundance of disulfide reductases in the ER which causes Prx4 disulfides to accumulate instead [18]. As well as the availability of disulfide reductases, the extent to which Prx become hyperoxidized *in vivo* will also be influenced by other aspects of its local environment, such as local H_2_O_2_ concentration, compartmental volumes and competition with other peroxidases. Here we have developed a computational model incorporating both the biochemical properties and *in vivo* environment as a tool to investigate when Prx become hyperoxidized and the effect of hyperoxidation on cell responses to H_2_O_2_. Notably, our model predicts that cell’s contain a Prx-independent H_2_O_2_-removing activity that becomes saturated following exposure to specific concentrations of extracellular H_2_O_2_. This results in a bi-phasic response to increased levels of ectopic H_2_O_2_ with Prx hyperoxidation only occurring once this H_2_O_2_–removing activity is saturated and intracellular H_2_O_2_ levels start to rise more rapidly. Importantly, we have experimentally confirmed these predictions in both yeast and human cells. As we discuss, our study provides new mechanistic insight into how Prx hyperoxidation and regulation of thioredoxin activity allow cells to implement appropriate responses to the different levels of H_2_O_2_ encountered *in vivo*.

## 2 Results

### 2.1 Quantitative analysis of how the *in vivo* oxidation of the Prx, Tpx1, changes in response to different concentrations of H_2_O_2_

To facilitate the generation of a mathematical model capable of simulating *in vivo* changes in Prx oxidation, we first obtained quantitative data for the *in vivo* oxidation of the single *S. pombe* peroxiredoxin Tpx1 following exposure of cells for 20 s to a range of H_2_O_2_ concentrations (0–6 mM) (Fig. 1 and Table S1). Western blot analysis of AMS-treated proteins using anti-Tpx1 antibodies detected multiple Tpx1-containing bands, as confirmed by the absence of these bands in Δ*tpx1* mutant cells (Fig. 1B). These included bands with mobilities of ~20 kDa and ~40 kDa consistent with representing reduced Tpx1 monomers (Tpx1SH) and Tpx1 disulfide dimers respectively. Under normal growth conditions the reduced monomeric band (Tpx1SH) was prevalent (Fig. 1B and Table S1). However, following treatment with H_2_O_2_, the levels of Tpx1SH decreased with the concomitant increased formation of disulfide dimers and higher molecular weight bands (>40 kDa), which likely include mixed disulfides with other proteins (Fig. 1B and data not shown). The sensitivity of all of these bands to reduction by beta-mercaptoethanol to 20 kDa forms (Fig. S1A) indicating that each represents a different disulfide-bonded form of Tpx1.

**Fig. 2:**
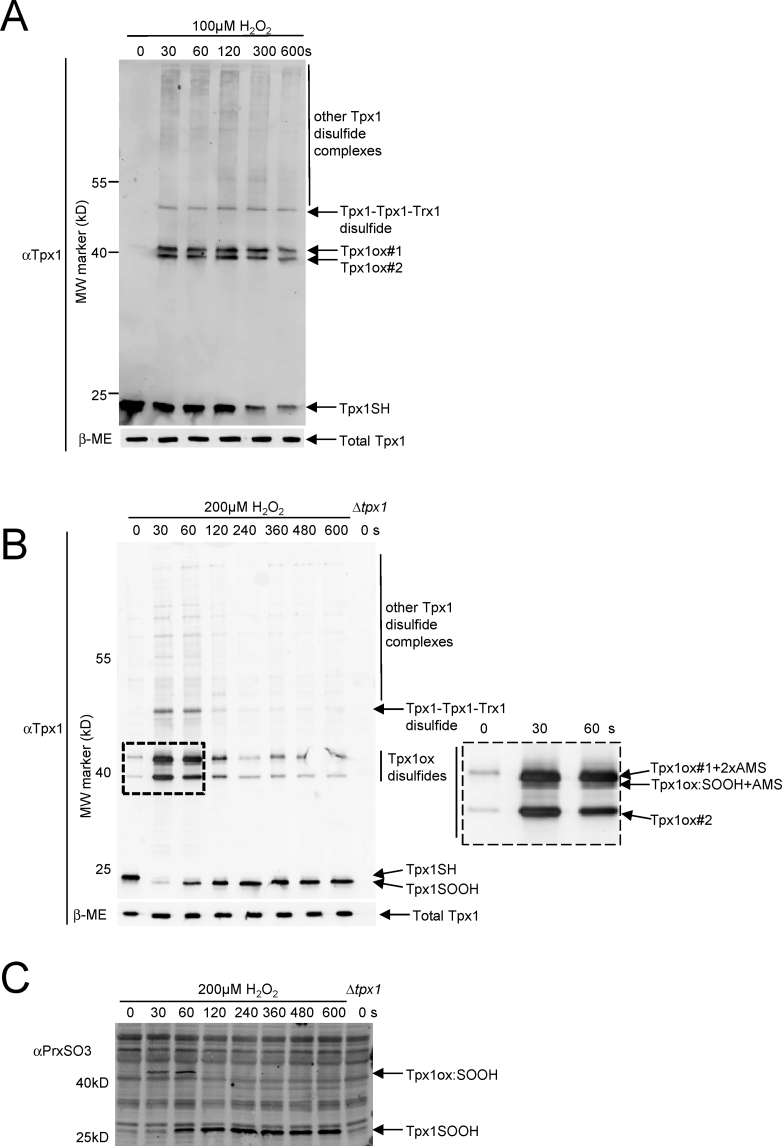
Changes in Tpx1 oxidation over time following treatment with 100 or 200 µM H_2_O_2_. Western blot analysis with (A) and (B) anti-Tpx1 or (C) anti-PrxSO3 antibodies of AMS-treated protein extracts from wild-type (972) and Δ*tpx1* mutant (VX00) cells treated, as indicated with (A) 100 µM (B) and (C) 200 µM H_2_O_2_ for 0–600 s shows how the oxidation of Tpx1 changes with time. (B) and (C) shows that Tpx1ox:SOOH disulfide formation at 200 µM H_2_O_2_ precedes the formation of Tpx1-SOOH monomers. In (B) a section of the blot, outlined by the dotted line, is magnified and shown in the right hand panel to enable the additional Tpx1ox form present in cells treated with 200 µM H_2_O_2_ (Tpx1ox:SOOH) to be seen more clearly. Western blot analysis of beta-mercaptoethanol (βME)-treated samples, run on a separate gel (lower panel in (A) and (B)), in which a single band represents all Tpx1 redox states (eliminating any influence that differences in mobility might have on transfer to the membrane) allowing total Tpx1 levels to be compared (A) confirms that differences between lanes reflect changes in Tpx1 oxidation rather than total Tpx1 levels (see also Table S1 and Fig. S2).

Although difficult to resolve, as expected, a magnified image revealed three distinct bands with mobilities consistent with Tpx1 disulfide homodimers (40 kDa) (Fig. 1C). The differences in the mobility of these three Tpx1 dimer bands was consistent with each representing a different redox state, as observed previously for Tpx1 and human Prx [15], [16], [19] (Fig. 1D). Indeed, in samples treated with NEM, which alkylates reduced cysteines producing a minimal increase in MW (0.1 kDa), a single Tpx1-containing band was detected at ~40 kDa (Fig. 1E). This confirms that the differences in the mobility of the 3 bands in AMS-treated samples reflect the different numbers of cysteine residues available to react with AMS, which increases the MW by 0.6 kDa per reduced cysteine. The single Tpx1 disulfide dimer band detected under normal conditions was significantly retarded by AMS. This is consistent with this band representing Tpx1–Tpx1 dimers containing a single disulfide bond between one peroxidatic (Cys_P_) and one resolving cysteine (Cys_R_) with the other cysteines reduced and AMS-reactive (Tpx1ox#1) (Fig. 1D and E). In cells treated with 200 µM H_2_O_2_ two additional Tpx1 disulfide bands were detected in the AMS-treated samples. The similar mobility of the lowest of these bands in NEM and AMS-treated samples suggests that it lacks any reduced cysteine thiols to react with NEM or AMS, consistent with it representing Tpx1 disulfide dimer containing two disulfide bonds (Tpx1ox#2) (Fig. 1D and E). The third band detected after treatment with 200 µM H_2_O_2_ (Tpx1ox:SOOH+AMS) had an intermediate mobility. This was consistent with the binding of a single AMS molecule to a single reduced cysteine thiol, as would be expected for Tpx1 disulfide dimers containing a single disulfide bond and a hyperoxidized Cys_P_ (Tpx1ox:SOOH) with the remaining Cys_R_ available to react with AMS (Fig. 1D and E). This form has also been detected using anti-PrxSO3 antibodies specific to the sulfinylated/sulfonylated peroxidatic cysteine [12]. Thus we confirmed that the three distinct Tpx1 disulfide dimers we detected represented redox states previously described for *S. pombe* and human peroxiredoxins [15], [16], [19]. In addition to the bands at 40 kDa, a band at 55 kDa was also detected (Figs. 1B, 2A, B and S1) that represents a Tpx1 dimer in a disulfide complex with Trx1 (Fig. S1B), a reaction intermediate in the reduction of Tpx1ox#2 by Trx1. Changes in the relative intensities of the various Tpx1 forms in cells treated with different H_2_O_2_ concentrations indicated that, as expected, Tpx1ox#1, Tpx1ox#2 and Tpx1ox:SOOH become the most prominent forms at the lowest, mid and high H_2_O_2_ concentrations respectively (Fig. 1 and Table S1).

### 2.2 Quantitative analysis of in vivo changes in the oxidation of Tpx1 with time following exposure to H_2_O_2_

To allow development of a dynamic model, we obtained kinetic data; determining how the relative abundance of different Tpx1 redox forms changed with time (≤600 s) following treatment with 100 µM or 200 µM H_2_O_2_. This time-course was selected to represent the initial H_2_O_2_ response, before H_2_O_2_-induced increases in mRNA levels [20], [21] have had any significant effect on total Tpx1 protein levels, or the levels of other proteins which might impact on Tpx1 oxidation [9], [10], [22] (Fig. 2 and data not shown). Tpx1ox#1 and Tpx1ox#2 were detected following treatment with 100 µM H_2_O_2_, but the relative levels of these forms did not change with time (Fig. 2A). Importantly, there was negligible formation of Tpx1ox:SOOH during the 100 µM time course (Table S1), as was confirmed using antibodies specific to hyperoxidized Tpx1 (Fig. S2). In contrast, as expected (Fig. 1) Tpx1ox#1, Tpx1ox#2 and Tpx1ox:SOOH dimers were all detected in cells treated with 200 µM H_2_O_2_ (Fig. 2B and C). However, the intensity of the Tpx1 dimer bands decreased over time with the concomitant formation of a Tpx1-containing monomeric band (~20 kDa). The increased mobility, compared with the AMS-reactive Tpx1-SH monomer band detected before addition of H_2_O_2_, suggests this H_2_O_2_-induced band represents monomeric Tpx1 containing a single AMS-reactive cysteine thiol. This is consistent with the hyperoxidized Tpx1SOOH monomer previously observed following treatment of cells with higher H_2_O_2_ concentrations [9], [10], [11], [12]. The formation of Tpx1-SOOH monomer was confirmed using antibodies specific to hyperoxidized Tpx1 (Figs. 2C and S2). The 200 µM time course data thus suggests that Tpx1ox:SOOH appears rapidly and is converted to Tpx1SOOH over time with the reaction essentially complete by 600 s. The detection of Prxox:SOOH dimers prior to PrxSOOH monomers is consistent with previous studies of Prx hyperoxidation [15], [19]. At 500 µM and 1000 µM H_2_O_2_ the monomeric Tpx1SOOH form was maximal by 60 s, suggesting that increased H_2_O_2_ concentration results in an increased rate of formation of Tpx1SOOH (Fig. S2). Importantly, consistent with published studies [9], [10], [22], western blot analysis of beta-mercaptoethanol treated samples confirmed that differences in the intensity of different Tpx1-containing bands did not reflect changes in total Tpx1 levels (Fig. 2A and B). Thus quantitative analysis of images, obtained for multiple independent biological repeats of these experiments (Fig. 1, Fig. 2), was used to estimate the concentrations of each Tpx1 oxidation state at each time point and level of H_2_O_2_ (Table S1).

### 2.3 A kinetic model of Tpx1 oxidation was developed that can replicate the experimental data

**Fig. 3:**
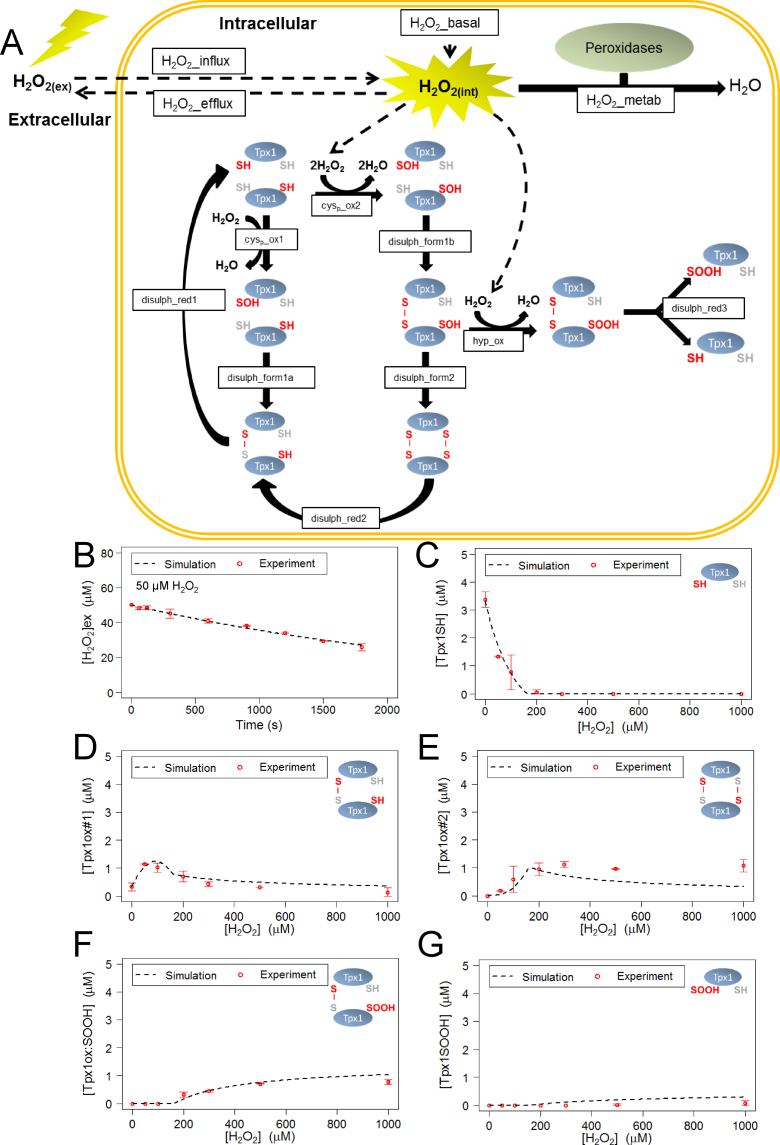
Diagram representing the mathematical model describing the *in vivo* oxidation of Tpx1 and qualitative analysis of the fit of the model to the experimental data: (A) the model contains 9 different Tpx1 oxidation states which are interconverted by the indicated reactions. The rates of influx/efflux (H_2_O_2_ influx and H_2_O_2_ efflux) and removal of H_2_O_2_ (H_2_O_2_ metab) from the intracellular compartment were also included in the model. For rate laws see Table S2 and for parameters see Table S3. (B)–(G) Plots show simulated and experimentally determined concentrations of (B) extracellular H_2_O_2_ (C) reduced Tpx1; Tpx1SH (D) single Tpx1 disulfides; Tpx1ox#1 (E) double Tpx1 disulfides; Tpx1ox#2 (F) disulfide bonded hyperoxidized Tpx1; Tpx1ox:SOOH (G) hyperoxidized Tpx1 monomer; Tpx1SOOH in wild-type *S. pombe* following 20 s exposure to between 0 and 1000 µM H_2_O_2_. Simulated data derived from the model were plotted against the experimental data used in the parameter estimation (Table S1) (see also Tables S2 and S3, Figs. S3 and S4).

**Fig. 4:**
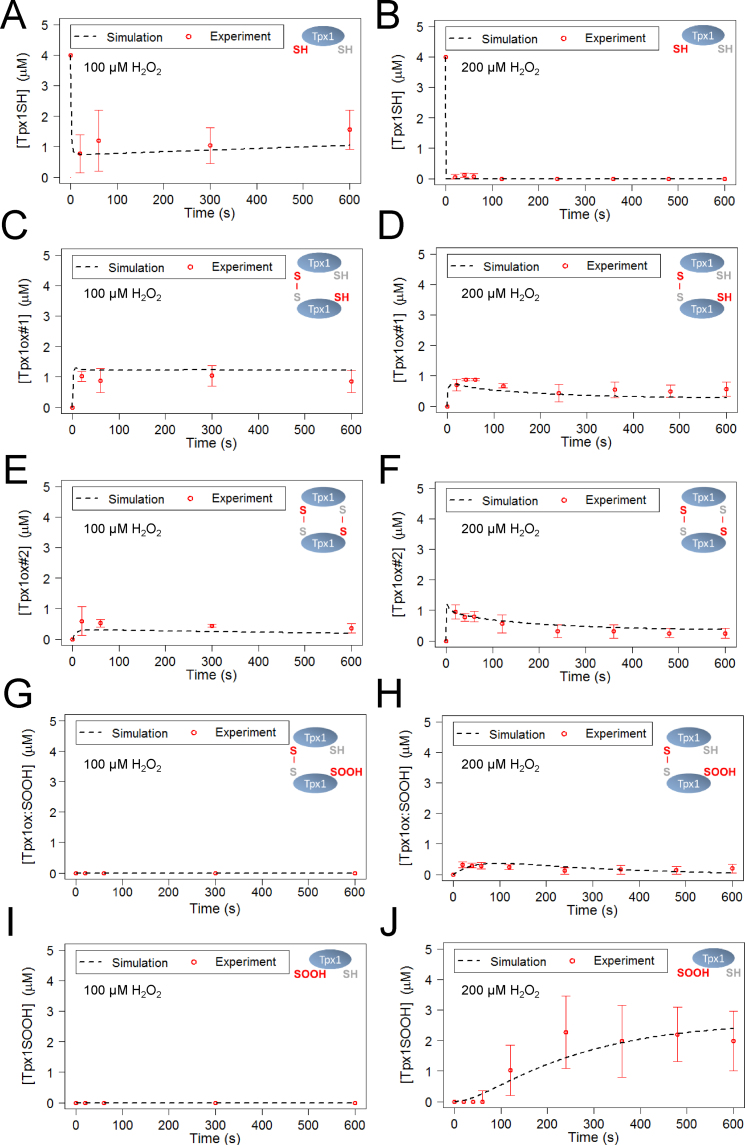
Qualitative analysis of the fit of the model to the experimental data for between 0 and 600 s exposure to 100 or 200 µM H_2_O_2_. Plots show simulated and experimentally determined concentrations of (A) and (B) reduced Tpx1; Tpx1SH (C) and (D) single Tpx1 disulfides; Tpx1ox#1 (E) and (F) double Tpx1 disulfides; Tpx1ox#2 (G) and (H) disulfide bonded hyperoxidized Tpx1; Tpx1oxSOOH (I) and (J) hyperoxidized Tpx1 monomer; Tpx1SOOH in wild-type *S. pombe* following 0–600 s treatment with (A), (C), (E), (G) and (I) 100 µM or (B), (D), (F), (H) and (J) 200 µM H_2_O_2_. Simulated data derived from the model were plotted against the experimental data used in the parameter estimation (Table S1).

Computational models to describe Tpx1 oxidation were then constructed using reaction networks and parameters that were selected based on published mechanisms, *in vitro* kinetic data, and our *in vivo* experimental data (Fig. 1, Fig. 2 and Tables S1–S3). We carried out qualitative and quantitative assessments of the ability of each model to describe the experimental data. This led to the selection of a final model, depicted in Fig. 3A, that used the parameter set in Table S3. This final model was able to simulate the removal of H_2_O_2_ from the extracellular space, indicating that this model could effectively represent the peroxide-removing activity of the cells (Fig. 3B). The final model also simulated the changes in the concentrations of the different Tpx1 redox states following 20 s exposure to H_2_O_2_ concentrations between 0 and 1000  µM (Fig. 3C–G) or up to 600 s following exposure to 100 or 200 µM H_2_O_2_ (Fig. 4). Importantly, the parameters that were predicted for this model were similar to published values (Table S3). For instance, the parameters *k_disulph_red1_*, *k_disulph_red2_* were predicted to be 0.190 and 0.143 µM^−1^ s^−1^ respectively (Table S3, Figs. S3 and S4), broadly consistent with experimentally determined values [23], [24]. This indicated that the model accurately represents the rapid Trx1-mediated reduction of the disulfide bonds in Tpx1ox#1 and Tpx1ox#2. Interestingly, the rate constant for the reduction of Tpx1ox:SOOH, *k_disulph_red3_*, was predicted, to be 0.029 µM^−1^ s^−1^, 5 fold lower than either *k_disulph_red1_* or *k_disulph_red2_* (Table S3, Figs. S3 and S4). This suggests that Trx1 may be less efficient at reducing the disulfide bond in Tpx1ox:SOOH compared with the other Tpx1 disulfide dimers. Notably, the first order rate constant for Tpx1 disulfide formation (rate constant *k_disulph_form2_*) estimated by our model as 3.44 s^−1^ was in a similar range to the rates of disulfide formation estimated for human Prx1 and Prx3 (2 s^−1^ and 20 s^−1^ respectively) from *in vitro* experimental investigations [16]. This supports the use of our model and *in vitro* studies as complementary tools to understand the *in vivo* oxidation of yeast and human Prx.

### 2.4 Modeling suggests that oxidation of the peroxidatic cysteine thiol (Cys_P_) in Tpx1ox#1 is not the major route for further oxidation of Tpx1

**Fig. 5:**
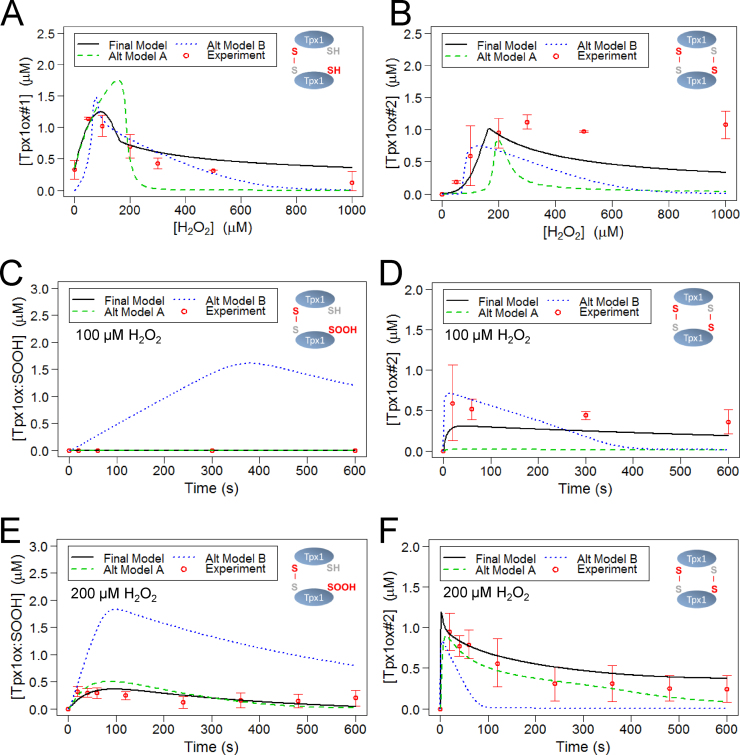
Alternative models, Alt Model A and Alt Model B, were unable to simulate the experimentally determined changes in Tpx1 oxidation state following treatment of wild-type *S. pombe* with H_2_O_2_ as effectively as the final model. All 3 models were constructed using the same reaction network (Fig. 3A) and rate laws (Table S2) except that in alternative model A, Tpx1ox:SOH was also produced by the H_2_O_2_-induced oxidation of Tpx1ox#1 in a reaction governed by the rate law *k_cys_ox3_* [Tpx1ox#1][H_2_O_2_]_int_. Alternative model B was identical with the final model except that it lacked the peroxide-removing reaction, H_2_O_2__metab. For parameters sets used in models see Tables S3 and S4. See also Table S5.

Having built and parameterized a dynamic model capable of simulating the *in vivo* oxidation of Tpx1 (Fig. 3, Fig. 4), we tested which aspects of our model were important for simulation of the experimental data by comparing the ability of our final model and 2 alternative models to simulate the dynamics of Tpx1 oxidation. Although difficult to demonstrate experimentally, it seemed likely that the reaction of the free Cys_P_ in Tpx1ox#1 with H_2_O_2_ to form Tpx1ox:SOH would be involved in the formation of both the Tpx1ox#2 disulfide dimer and Tpx1ox:SOOH. However, unexpectedly, alternative Model A which included this additional reaction (Table S4) was less able to simulate the experimentally observed dynamics of Tpx1 oxidation (Fig. 5) and had an increased AIC compared with the final model (Table S5). Accordingly, this alternative model was rejected in favor of our final model (Fig. 3A) in which the simultaneous oxidation of neighboring Cys_P_-SH (disulph_form1b) is the major route of Tpx1ox:SOH formation. Interestingly, this suggests that the reduced Cys_P_ in Tpx1ox#1 may be less sensitive to H_2_O_2_ and that, *in vivo*, the reaction of neighboring peroxidatic cysteines with H_2_O_2_, prior to disulfide bond formation, may be prerequisite for the generation of hyperoxidized SOOH derivatives.

Although an alternative route for the formation of hyperoxidized Tpx1 would be via the oxidation of the sulfenylated peroxidatic cysteine in Tpx1-SOH, the inclusion of this reaction was not required to fit the experimental data, and actually rendered models less able to simulate the experimentally observed dynamics of Tpx1 oxidation (data not shown). Thus, we conclude that this route makes a negligible contribution to the pool of hyperoxidized Tpx1SOOH detected at these H_2_O_2_ concentrations. This is consistent with previous work [19] suggesting that the hyperoxidation of Tpx1ox:SOH precedes the formation of Tpx1SOOH disulfide dimers and is the dominant route for Tpx1 sulfinylation *in vivo*.

### 2.5 The removal of H_2_O_2_ by an additional process/es (H_2_O_2__metab) was required to explain the dynamics of Tpx1 hyperoxidation

The final model (Fig. 3A) accurately simulated our experimental observation that there was negligible formation of either of the hyperoxidized Tpx1 forms (Tpx1ox:SOOH and Tpx1-SOOH) at H_2_O_2_ concentrations below 200 µM (Figs. 1, 4 and S2). However, this particular feature of the experimental data was not captured by alternative Model B which did not contain the reaction ‘H_2_O_2__metab’ (Table S4). Instead, alternative Model B predicted that Tpx1 would be hyperoxidized at all H_2_O_2_ concentrations, even below 200 µM (Alt Model B Fig. 5C and E). This suggests that, although Tpx1 reacts with extremely low H_2_O_2_ concentrations to generate Tpx1–Tpx1 disulfides, other components of the cell’s H_2_O_2_-buffering capacity normally inhibit hyperoxidation *in vivo*. Together these results suggest that the sensitivity of peroxiredoxins to hyperoxidation is not just dependent on the kinetics of Prx reaction with H_2_O_2_ but is also heavily influenced by the other peroxide-reactive molecules present in their *in vivo* environment.

### 2.6 As predicted by our model, there is a two phase relationship between intracellular and extracellular H_2_O_2_ concentration

**Fig. 6:**
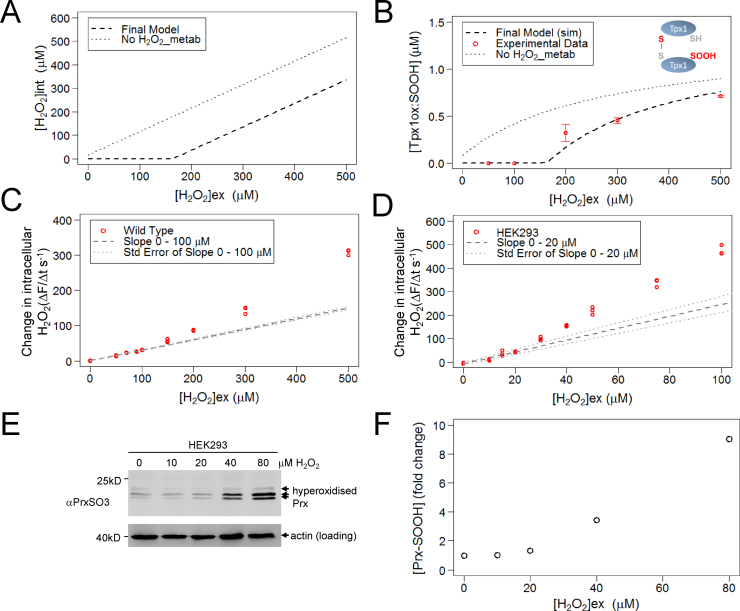
As predicted by the model, hyperoxidation of Prx only occurs when the H_2_O_2_-removing capacity of cells is saturated. (A) and (B) The effect of increasing extracellular H_2_O_2_ on (A) intracellular H_2_O_2_ concentration (B) hyperoxidation of Tpx1 was simulated using the final model with the rate constant *V_max_H2O2_metab_* set as 59  µM s^−1^ (final model) or 0 µM s^−1^ (No H_2_O_2__metab). In (B) the experimentally determined effects of increasing extracellular H_2_O_2_ concentrations on the hyperoxidation of Tpx1 are also shown. (C) and (D) Experimental measurements using a fluorescent H_2_O_2_-specific dye (PF3) (25) of the rate at which intracellular H_2_O_2_ increases (Δ*F*/Δ*t*) in (C) wild-type *S. pombe* (972) (D) human cells (HEK293) following exposure to increasing concentrations of H_2_O_2_. The results of three independent experiments are shown. The gradient (m±SE) and intercept (c±SE) were calculated using data points (C) 0–100 μM (*S. pombe*) (D) 0–20 μM (HEK293) and extrapolated to create the “Slope” and “Std. Error of Slope” lines that are shown. (E) and (F) The increase in hyperoxidized Prx in HEK293 cells treated with increasing concentrations of H_2_O_2_ was determined experimentally by (E) western blotting with anti-PrxSO3 antibodies of proteins extracted from HEK293 cells following 10 min exposure to the indicated concentration of H_2_O_2_. This revealed an increase in beta-mercaptoethanol-resistant hyperoxidized Prx (indicated by the arrows) which were (F) quantified as total hyperoxidized Prx relative to a loading control (actin).

To further investigate the conditions which cause Prx to become hyperoxidized, we used our model to predict how increasing extracellular H_2_O_2_ concentrations ([H_2_O_2_]_ex_) would affect the intracellular H_2_O_2_ concentration ([H_2_O_2_]_int_) (Fig. 6A). Interestingly, the final model predicted that [H_2_O_2_]_ex_<100  µM would cause little net change in [H_2_O_2_]_int_ but that at [H_2_O_2_]_ex_>150 µM the [H_2_O_2_]_int_ increases linearly with increasing [H_2_O_2_]_ex_ (Fig. 6A). This two phase relationship between intracellular and extracellular [H_2_O_2_] was dependent on the reaction H_2_O_2__metab (Figs. 3A and 6A). This reaction, absent from alternative Model B, represents the cell’s other peroxidase activities and is required for the model to accurately simulate the dynamics of Tpx1SOOH formation (Figs. 5C, E and 6B). Thus our model predicts that this peroxidase activity is saturated following treatment of wild-type cells with 150 µM H_2_O_2_. To test this prediction we used the H_2_O_2_ sensitive fluorescent dye, PF3, to measure the rate at which [H_2_O_2_]_int_ increases in cells following exposure to different [H_2_O_2_]_ex_ [25]. Although we detected a steady increase in the accumulation of intracellular H_2_O_2_ in cells exposed to low concentrations of H_2_O_2_, as predicted by our model, the rate of intracellular H_2_O_2_ accumulation was much faster in cells exposed to extracellular H_2_O_2_ concentrations greater than 150 µM (Fig. 6C). Notably, as predicted by the model, and experimentally confirmed, significant formation of hyperoxidized Tpx1 (Tpx1ox:SOOH) only begins to occur following exposure of cells to similar [H_2_O_2_]_ex_ to those saturating H_2_O_2__metab (Fig. 6B). Having established that our model was able to make accurate predictions in *S. pombe* we examined whether these findings held true in human cells.

To test whether there was also a biphasic effect of increasing [H_2_O_2_]_ex_ on the rate at which intracellular H_2_O_2_ levels increase in human cells, we examined how exposure to different extracellular concentrations of H_2_O_2_ affected the intracellular H_2_O_2_ concentration in human embryonic kidney (HEK293) cells. A lower level of extracellular H_2_O_2_ was required to breach the H_2_O_2_-buffering capacity of HEK293 cells than *S. pombe*, possibly reflecting the increased H_2_O_2_-permeability of human cells (Fig. 6C and D). Nevertheless, we observed a similar 2 phase relationship between extracellular and intracellular H_2_O_2_ concentrations; human cells were able to maintain a low intracellular H_2_O_2_ concentration following exposure to lower concentrations of H_2_O_2_ but once this buffering capacity was exceeded (≥40 μM) the intracellular H_2_O_2_ concentration rose more rapidly (Fig. 6D). Furthermore, using anti-PrxSO3 antibodies that recognize the hyperoxidized forms of all 4 human 2-Cys Prx [18], [26], we found that, similar to our findings in yeast (Fig. 6B), the *in vivo* hyperoxidation of human Prx only began to increase once the H_2_O_2_-buffering capacity of HEK293 cells was overwhelmed and intracellular H_2_O_2_ levels started to increase more rapidly (Fig. 6E and F). Together these data reveal that there is a biphasic increase in intracellular H_2_O_2_ in response to increases in extracellular H_2_O_2_ in yeast and human cells, with cells able to buffer exposure to low levels of extracellular H_2_O_2_ more effectively. Moreover, these data are consistent with the model’s prediction that saturation of this buffering capacity triggers the hyperoxidation of Prx.

### 2.7 Hyperoxidation of Prx detects the point at which other peroxide-removing processes (H_2_O_2__metab) become saturated

**Fig. 7:**
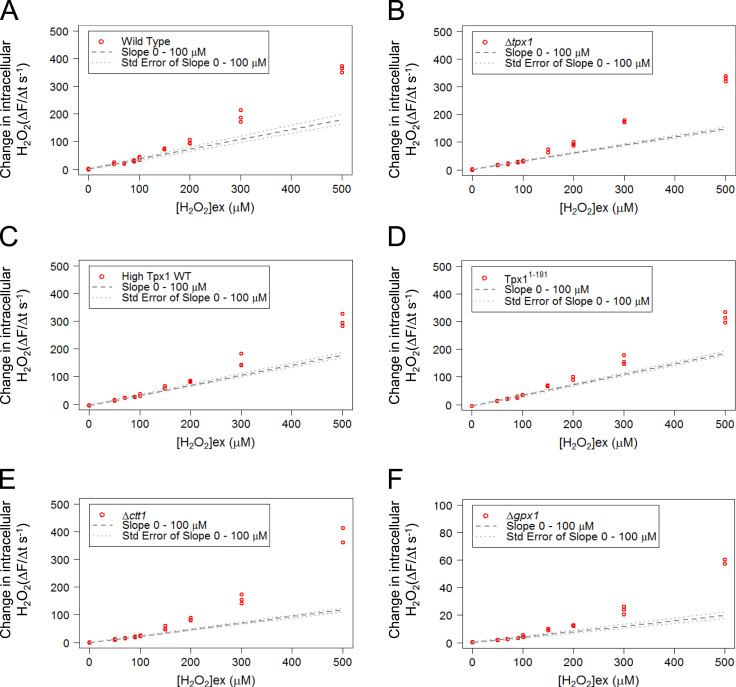
Tpx1, Gpx1 and catalase activity, do not influence the biphasic increase in intracellular H_2_O_2_ in response to increasing extracellular H_2_O_2_. The rate of intracellular H_2_O_2_ accumulation, measured using a fluorescent H_2_O_2_-specific dye (PF3) [25], following exposure of (A) wild type (NT4) (B) Δ*tpx1* (VX00) mutant (C) cells expressing higher levels of wild-type Tpx1; High Tpx1 WT (JR68) or (D) truncated, hyperoxidation-resistant Tpx1; Tpx1^1-181^ (JR20) (E) Δ*ctt1* (LT3) (F) Δ*gpx1* (SB13) to a range of H_2_O_2_ concentrations. The results of three independent experiments are shown. The gradient (m±SE) and intercept (c±SE) for the data points 0–100 µM were calculated and extrapolated to create the lines “Slope 0–100 µM” and “Std. Error of Slope 0–100 µM” (see “Methods”).

Accordingly, based on these modeling and experimental approaches, we propose that Prx hyperoxidation only occurs *in vivo* once the cell’s peroxide-removing capacity becomes saturated and intracellular H_2_O_2_ levels start to increase more rapidly. This hypothesis, is consistent with *in vitro* work demonstrating that catalase is able to specifically inhibit the hyperoxidation of human Prx1 [16]. However, it was possible that the increased accumulation of H_2_O_2_ observed in cells exposed to higher [H_2_O_2_]_ex_ e.g. >150 µM H_2_O_2_ in *S. pombe* (Fig. 6C) was due to the coincident hyperoxidation of Prx to peroxidase-inactive forms, rather than the saturation of other cellular peroxidases, To test this possibility we began by examining the effect of increasing [H_2_O_2_]_ex_ on intracellular H_2_O_2_ in Δ*tpx1* mutant *S. pombe*. Notably, loss of Tpx1 had little effect on the H_2_O_2_-buffering capacity of cells (Fig. 7A and B). Furthermore, the peroxide-buffering capacity of cells expressing increased levels of either wild-type Tpx1 or a Tpx1 isoform that is 10× more resistant to hyperoxidation [12], [27], was also saturated following exposure to 150 µM H_2_O_2_ (Fig. 7C and D). Together these data strongly suggest that the increase in the rate of H_2_O_2_ accumulation in cells exposed to [H_2_O_2_]_ex_>150 µM is due to the saturation of other cellular peroxide-buffering processes, rather than the inactivation of the thioredoxin peroxidase activity of Tpx1. Instead, this suggests that the hyperoxidation of Tpx1 *in vivo* actually detects the point at which the H_2_O_2_ buffering capacity of the cell is overcome and intracellular levels of H_2_O_2_ begin to rise more rapidly.

### 2.8 Thioredoxin is required to buffer low levels of extracellular H_2_O_2_

Having established that Tpx1 activity did not make an important contribution to the cell’s ability to maintain low intracellular H_2_O_2_ levels following exposure to exogenous H_2_O_2_, we next investigated which other enzyme/s might be responsible for this H_2_O_2_-buffering capacity. Catalase has previously been demonstrated to protect Prx against hyperoxidation *in vitro* [16]. However, although Ctt1 appears to limit the increase in intracellular H_2_O_2_ at higher concentrations of H_2_O_2_ (Fig. 7E), the similar 2 phase relationship between extracellular and intracellular H_2_O_2_ concentrations in Δ*ctt1* mutant cells suggests that catalase does not make an important contribution to the cell’s ability to buffer low levels of H_2_O_2_<150 µM (Fig. 7E). It was possible that the Gpx/GSH system [28], or other subfamilies of Prx, contributed to the H_2_O_2_-buffering capacity of *S. pombe*, this seemed unlikely given the minimal effect that deletion of the genes encoding these enzymes (*gpx1*, *pmp20* or *dot5*) has on H_2_O_2_ resistance [10]. Indeed, loss of Gpx1 had no effect upon the bi-phasic relationship between extracellular and intracellular H_2_O_2_ (Fig. 7F).

**Fig. 8:**
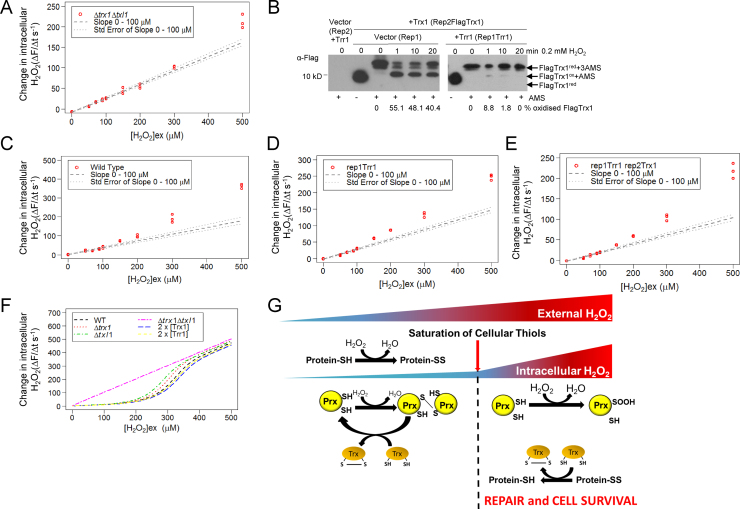
Thioredoxin is required for the biphasic increase in intracellular H_2_O_2_ in response to increasing extracellular H_2_O_2_, consistent with the oxidation of protein-thiols limiting rises in intracellular H_2_O_2_ following exposure of *S. pombe* to low levels of extracellular H_2_O_2_ (A), (C)–(E) the rate of intracellular H_2_O_2_ accumulation, using a fluorescent H_2_O_2_-specific dye (PF3) [25], following exposure of (A) Δ*trx1*Δ*txl1* (AD140) (C)–(E) wild type (NT4) cells containing (C) vector control (Rep1) (D) and (E) ectopically expressing additional Trr1 (Rep1Trr1) [11] and (E) Flag-tagged Trx1 (Rep2Trx1), to a range of H_2_O_2_ concentrations. (B) Western blot analysis of FlagTrx1 redox state in cells containing vector control or overexpressing Trr1 (Rep1Trr1) before and following exposure for the indicated times to 0.2 mM H_2_O_2_. Image J quantification was used to determine the % FlagTrx1 oxidation. (F) The oxidation of protein thiols (Pr-SH) to protein disulfides (Pr-SS) with a low affinity for thioredoxin (Trx1/Txl1) is able to explain the H_2_O_2_-buffering capacity that is saturated following exposure to 150 µM H_2_O_2_. In this model (Tables S6 and S7) increasing oxidoreductase activity, by doubling the concentrations of Trx1 or Trr1, produces a negligible effect on the H_2_O_2_-buffering capacity, whereas loss of both Trx1 and Txl1 ablates the H_2_O_2_-buffering capacity. (G) We propose that reduced protein thiols (Protein-SH), act like a sponge, contributing to a buffer that prevents increases in intracellular accumulation of H_2_O_2_ but that, following maximal oxidation of these cellular protein-thiols to disulfides (Protein-SS), intracellular H_2_O_2_ concentrations increase, causing peroxidatic cysteines in Prx to become hyperoxidized to sulfinic derivatives (SOOH) and diverting thioredoxin towards the reduction of other protein disulfides. See also Tables S6, S7 and Fig. S5.

Our previous studies have established that treatment with 0.2 mM H_2_O_2_ causes the majority of Trx1 and Txl1 to become rapidly oxidized [11], [12]. As broad specificity oxidoreductases, thioredoxin family proteins are important cofactors for many enzymes and for the reduction of other oxidized proteins that may also impact on intracellular H_2_O_2_ levels [12], [29], [30]. Although, Tpx1 and Gpx1 were not important for buffering low levels of H_2_O_2_ (Fig. 7), it was still possible that the point at which the H_2_O_2_-buffering capacity is breached might reflect the point at which thioredoxin reductase activity becomes limiting for the removal of H_2_O_2_ by other thioredoxin-dependent activities. Hence, to test whether thioredoxin-dependent processes are important for inhibiting increases in intracellular H_2_O_2_ in cells exposed to ≤150 μM H_2_O_2_, we examined how extracellular H_2_O_2_ treatment affected intracellular H_2_O_2_ concentration in mutant *S. pombe* lacking, or ectopically expressing additional thioredoxin and/or Trr1. Strikingly, the bi-phasic relationship between extracellular [H_2_O_2_] and the rate of increase in intracellular [H_2_O_2_] was lost in Δ*trx1*Δ*txl1* cells in which both thioredoxin family proteins are absent (Fig. 8A). This linear relationship between intracellular and extracellular H_2_O_2_ concentrations up to 500 μM H_2_O_2_ in Δ*trx1*Δ*txl1* mutant cells indicated that the peroxide-removing activity that is normally saturated by low levels of H_2_O_2_ (H_2_O_2__metab) requires thioredoxin (Fig. 8A).

Accordingly, we tested whether the point at which the H_2_O_2_-buffering capacity is breached might reflect the point at which thioredoxin reductase activity becomes limiting for the removal of H_2_O_2_ by thioredoxin-dependent processes. Importantly, overexpressing Trr1 prevented the rapid and sustained oxidation of Trx1 in cells treated with 0.2 mM H_2_O_2_, indicating that thioredoxin reductase activity, rather than NADPH levels, normally limits Trx1 reduction under these conditions (Fig. 8B). However, overexpressing Trx1 and/or Trr1 had a negligible effect on the relationship between extracellular [H_2_O_2_] and the rate of increase in intracellular [H_2_O_2_] (Fig. 8C–E). This suggests that the saturation of the cell’s H_2_O_2_-buffering capacity is not due to the saturation of thioredoxin reductase activity.

Computational modeling and experimental studies have suggested that, although most protein thiols are relatively insensitive to oxidation, the reversible oxidation of H_2_O_2_-sensitive protein cysteine-thiols (PSH) (the thiol proteome) might still make an important contribution to the peroxide-buffering capacity of human cells [31], [32]. Indeed, it has been estimated that the concentration of oxidant accessible protein thiols is around 13 mM, similar to the total concentration of glutathione [32]. Although most cellular thiols have a much lower reactivity with H_2_O_2_ than Prxs [33], the shear abundance of these H_2_O_2_-reactive thiols could mean that collectively they make a large contribution to the H_2_O_2_-buffering capacity of the cell. Moreover, previous work has shown that, like Trx1 and Txl1, the thiol proteome is also maximally oxidized following exposure of *S. pombe* to 200 μM H_2_O_2_ [29]. Hence, by causing constitutive, maximal oxidation of the thiol proteome [29], the absence of both cytosolic thioredoxin family proteins, Trx1 and Txl1, could potentially ablate the H_2_O_2_-buffering ability of this thiol pool. To test whether, despite their lower reactivity, the intracellular concentration of H_2_O_2_-oxidizable protein-thiols could be sufficient to make a significant contribution to the cell’s capacity to buffer H_2_O_2,_ we constructed a simple computer model, assuming 13 mM protein thiols with an average reactivity with H_2_O_2_ of 0.0005 µM^−1^ s^−1^. This model was able to simulate the *in vivo* bi-phasic response in intracellular H_2_O_2_, illustrating the concept that the oxidation of 13 mM reduced protein-thiols (Tables S6 and S7), could prevent rises in intracellular H_2_O_2_ following treatment with up to 0.2 mM H_2_O_2_ (Fig. 8F). This model also predicted that, provided the thioredoxin-reactivity of the resulting protein disulfides is low, the initial concentration of reactive thiols, will have a greater influence on the buffering capacity than the availability of thioredoxin (Trx1) (Figs. 8F and S5). Accordingly, this model simulates the negligible effect of overexpressing Trx1 and/or Trr1 on the relationship between extracellular [H_2_O_2_] and the rate of increase in intracellular [H_2_O_2_] (Fig. 8C–F). This is consistent with the idea that the initial oxidation of protein thiols, rather than other thioredoxin-dependent enzymatic processes, could be responsible for the observed buffering of low levels of external H_2_O_2_ (Figs. 7 and 8C–E). Importantly, our model also recapitulated the effect of loss of Trx1 and Txl1 on intracellular H_2_O_2_ (Fig. 8A and F). Given that systems of redox-couples have previously been demonstrated to display ‘apparent’ Michaelis–Menten kinetics [34], [35], this suggests that the *V_max_* of the H_2_O_2__metab, predicted in our model (Fig. 3 and Table S3), could represent the apparent *V_max_* for removal of H_2_O_2_ by oxidation of the thiol proteome. Clearly the representation of the diverse protein-thiol pool in our model is a gross oversimplification and we note that the average reactivity we use requires a larger pool of protein thiols to react rapidly with H_2_O_2_ than might be expected from *in vitro* studies. Hence, although our experimental data indicate that thioredoxin is vital (Fig. 8A), it is possible that H_2_O_2_-reactants, such as glutathione or methionine, not investigated here, also make important contributions to the cell’s capacity to buffer the intracellular environment against rises in extracellular H_2_O_2_. Nevertheless, our model and data are consistent with other studies that have suggested that the oxidation of the thiol proteome makes a major contribution to the *in vivo* removal of H_2_O_2_ (Fig. 8) [31], [32]. Importantly, here we show that the H_2_O_2_-induced hyperoxidation of Prx only occurs once this peroxide-buffering capacity is saturated (Figs. 6 and 8G). Based on these findings, we propose that hyperoxidation is a response to increased intracellular H_2_O_2_, allowing downstream signaling, as well as protective chaperone functions of hyperoxidized peroxiredoxin [8], [14]. These findings are also consistent with our previous studies which indicated that peroxiredoxin hyperoxidation is important for thioredoxin-mediated repair and cell survival (Fig. 8G) [12].

## 3 Discussion

The role of peroxiredoxins in cell responses to H_2_O_2_ has come under considerable scrutiny in recent years since the discovery that the thioredoxin peroxidase activity of Prx is sensitive to inactivation by H_2_O_2_. Several functions have been proposed for the H_2_O_2_-induced inactivation of this peroxidase activity. However, to assess if/when any or all of these functions are important, it is important to understand under which circumstances Prx become hyperoxidized (inactivated) *in vivo*. Here we have developed a mathematical model describing the kinetics of oxidation of the single *S. pombe* 2-Cys Prx in response to H_2_O_2_ as a tool to investigate the precise circumstances that cause Prx to become hyperoxidized *in vivo*. This model has made several unexpected predictions.

Firstly, our model suggested that there is a biphasic relationship between extracellular and intracellular H_2_O_2_ such that exposure to low levels of H_2_O_2_ produces only small increases in the intracellular H_2_O_2_ concentration, whereas above a certain threshold the cell’s peroxide-removing capacity becomes overwhelmed and intracellular H_2_O_2_ concentrations increase at a much faster rate. Importantly, this prediction was experimentally confirmed in both yeast and human cells.

Secondly, our model reveals that this peroxide-removing activity protects Tpx1 from hyperoxidation. This explains why hyperoxidized Tpx1 is only detected following exposure to extracellular concentrations of H_2_O_2_ above 100 µM. Notably, this is consistent with work in mammalian cells, which can effectively buffer extracellular H_2_O_2_ concentrations of 10 μM that cause some hyperoxidation of Prx2 *in vitro* [16], [36] but which require higher extracellular H_2_O_2_ concentrations to increase intracellular H_2_O_2_ levels and also cause *in vivo* hyperoxidation of Prx [6], [36], [37]. Indeed, not only do we confirm that the biphasic relationship between extracellular and intracellular H_2_O_2_ also holds true in HEK293 cells, but also that hyperoxidation of human Prx only occurs once the ability of these cells to buffer H_2_O_2_ becomes saturated (Fig. 6D–F).

Thirdly, our modeling suggests that the main route for formation of the hyperoxidized Prx involves reaction of 2 neighboring catalytic centers (Cys_P_) with H_2_O_2_ prior to formation of Prx disulfides. Interestingly, this suggests that the reduced Cys_P_ in Tpx1ox#1 may be less sensitive to H_2_O_2_ than that in reduced Tpx1. This still needs to be experimentally validated, but could be explained by the propensity of disulfide formation to destabilize Prx decamers resulting in Prx dimers which are approximately 100 fold less reactive with H_2_O_2_ [38], [39]. Intriguingly, this would provide an explanation as to why Prx hyperoxidation only occurs once the peroxide-buffering capacity of the cell is saturated, as the more rapid increase in intracellular H_2_O_2_ concentration would greatly increase the probability of neighboring Cys_P_ reacting with H_2_O_2_ prior to disulfide formation.

Fourthly, our modeling and experimental investigations together indicate that the peroxide-removing processes that are saturated in *S. pombe* are likely to include the reversible oxidation of the thiol proteome. Notably, although the H_2_O_2_-buffering capacity predicted by our model is eliminated in the absence of disulfide reductase activity, loss of Trx1 alone had little effect upon the ability of cells to buffer intracellular H_2_O_2_ (data not shown). Moreover, ectopically overexpressing Trr1 and Trx1 did not increase the intracellular H_2_O_2_-buffering capacity (Fig. 8C–E). This suggests that the maximum rate of H_2_O_2_-removal is independent of the availability of these disulfide reductases. Instead, this suggests that the limiting factor for H_2_O_2_-buffering is the initial availability of reduced protein thiols, with the low affinity of these protein disulfides for Trx1/Txl1 limiting the rate at which they are regenerated. It has been estimated that the concentration of oxidant accessible protein thiols is around 13 mM, similar to the total concentration of glutathione [32]. Although it is possible that other activities, such as glutathione and methionine oxidation, also contribute, this is consistent with the saturation of this abundant pool of free protein-thiols making the most important contribution to the saturable H_2_O_2_ buffering capacity revealed by our computer model.

In response to 200 μM H_2_O_2_ both thioredoxin family proteins (Trx1 and Txl1) are completely oxidized in the reduction of Tpx1–Tpx1 disulfides [11], [12]. This Tpx1-dependent inhibition of Txl1, allows the sustained activation of the Pap1 transcription factor which promotes the expression of a host of oxidative stress defense enzymes [11]. However, thioredoxin (Trx1) is also vital for the reduction of oxidized protein cysteine-thiols and the activity of methionine sulfoxide reductase enzymes. Indeed, the thiol proteome is maximally oxidized in cells where Trx1 and Txl1 are inhibited genetically, or as a result of Tpx1-dependent oxidation [29]. Hence, it is logical that, once reactive protein thiols have fully reacted with H_2_O_2_, it is important to target thioredoxin activity away from Prx disulfides, for which they have much greater affinity, towards reducing these oxidized cysteine and methionine residues. Hyperoxidized Prx cannot be reduced by thioredoxin. Accordingly, it has been proposed that, by converting Prx to a form that is no longer a thioredoxin substrate, Prx hyperoxidation enables thioredoxin to be targeted to other oxidized proteins instead [12]. Interestingly, our model predicts that Tpx1 disulfides are less efficiently reduced by thioredoxin if the non-bonded Cys_P_ is sulfinylated (Tpx1ox:SOOH). It is possible that, under conditions where thioredoxin reductase is limiting, this may also help redirect thioredoxin activity towards other substrates, for which it has a much lower affinity. Indeed, consistent with these findings, the hyperoxidation of Tpx1 is important to maintain thioredoxin activity, allowing the repair of oxidized proteins, and cell survival following exposure of cells to higher concentrations of H_2_O_2_ [12].

The high H_2_O_2_-scavenging activity of Prx, such as Tpx1, when recycling systems are provided in excess *in vitro*, supports previous reports suggesting that the thioredoxin peroxidase activity of Tpx1 is important for removing the low levels of endogenous H_2_O_2_ generated during normal aerobic growth and metabolism [16,40]. However, here we show that this thioredoxin peroxidase activity makes a negligible contribution to *S. pombe*’s capacity to buffer the internal environment against extracellular increases in H_2_O_2_ (Fig. 7A–D). Perhaps this is not surprising given that under these *in vivo* conditions thioredoxin reductase activity is limiting, preventing the efficient recycling of Tpx1 disulfides (Fig. 8B) [12]. Indeed, our previous work, has suggested that, rather than its H_2_O_2_-detoxifying capacity, the important role of the thioredoxin peroxidase activity of Tpx1 in cells exposed to these levels of exogenous H_2_O_2_ is to promote the oxidation of Txl1 and hence H_2_O_2_-induced gene expression and oxidative stress resistance [11]. Consistent with this, a model of Prx2 oxidation in human erythrocytes has also suggested that the abundance and peroxidase activity of the Prxs favors a signaling rather than a peroxide-detoxification role [41].

The hyperoxidation of 2-Cys Prx has been identified as a conserved feature of circadian rhythms in eukaryotes [42], [43], [44] (for a review see [7]). Our model suggests that hyperoxidation only occurs when the cell’s capacity for H_2_O_2_-removal is breached, allowing the concerted reaction of 2 H_2_O_2_ molecules with adjacent peroxidatic cysteines. This raises the possibility that the hyperoxidized Prx detected in each of these organisms reflects a transient, daily increase in the intracellular H_2_O_2_ concentration above the cell’s peroxide-buffering capacity. Consistent with the possibility that a cyclic increase in ROS might be important for circadian rhythms, Nrf2, the transcription factor controlling the levels of peroxidase-removing enzymes in mammals, was recently shown to be regulated in a circadian pattern [45]. If an increase in intracellular H_2_O_2_ is important for circadian control of cellular activities, then it is possible that loss of this regulation may contribute to the deleterious effects that can be associated with increased dietary antioxidants and constitutively activated stress defenses.

The inactivation of 2-Cys Prx by hyperoxidation has been proposed to allow H_2_O_2_ to act as a signal [5]. However, where the amount of hyperoxidized has been compared with the total Prx, only a small proportion of the total pool of 2-Cys Prx appears to be hyperoxidized under normal growth conditions/during circadian rhythms [6], [8], [46]. Moreover, hyperoxidation of Prx is undetectable in response to the low H_2_O_2_ levels produced in response to growth factor activated NADPH oxidases [6], [37]. As 2-Cys Prx are highly abundant, and only 1 of a repertoire of peroxidase enzymes, it has seemed unlikely that inactivation of a small proportion would significantly impact on intracellular H_2_O_2_ levels. Indeed, as predicted by our model, our experimental data suggests that the complete inactivation of Tpx1, either by deletion or hyperoxidation, has minimal effect on *S. pombe*’s ability to prevent the intracellular accumulation of H_2_O_2_ (Fig. 7). Instead, our model is consistent with other work suggesting that hyperoxidation of Prx may have other functions in signaling or protein homeostasis [12], [14], [16], [47], [48].

In summary, our study provides new insight into the underlying causes and function of Prx hyperoxidation. Moreover, the discovery that extracellular increases in H_2_O_2_ produce non-linear increases in intracellular levels, which are dependent upon the levels of thioredoxin activity, has important implications for the host of studies which have used a bolus of H_2_O_2_ either as a stress or signaling stimulus. Indeed, the model we have developed provides an important new tool to predict responses to altered redox conditions. For example, our model reveals how differences between the thioredoxin or Prx activity in individual cells could precisely tailor the sensitivity/response of specific cells within a population to H_2_O_2_ signals and oxidative stress. Therefore, this has important implications for how dynamic redox changes initiate changes in cell function and behavior during normal physiology and in disease.

## 4 Materials and methods

### 4.1 Cell culture conditions

The *S. pombe* strains (Table S8) and human kidney (HEK293) cells used in this study were maintained using standard media and growth conditions. For experiments, *S. pombe* were grown with agitation at 30 °C in 50 ml Edinburgh minimal media (EMM) supplemented with 0.48 mM histidine, 0.56 mM adenine, 0.67 mM uracil, 1.91 mM leucine. In experiments involving Δ*trx1*Δ*txl1* cells, which are auxotrophic for cysteine, media was also supplemented with 0.52 mM cysteine. HEK293 cells were grown in a humidified CO_2_ incubator at 37 °C in 24 ml Dulbecco’s Modified Eagles Medium (DMEM) supplemented with 10% (v/v) fetal calf serum (FCS), 100 units/ml penicillin, 100 µg/ml streptomycin, 2 mM l-glutamine and 1% non-essential amino acids (NES).

### 4.2 Quantitative measurement of Prx or FlagTrx1 oxidation

*S. pombe*: 50 ml cultures of exponentially growing *S. pombe* (OD 0.4–0.5) were harvested before and after exposure to a range of H_2_O_2_ concentrations. At specific time points following addition of H_2_O_2_, 3 ml of culture (2.4–4.0×10^7^ cells) was harvested by adding an equal volume of 20% Trichloroacetic acid (TCA). Protein extracts were prepared essentially as described previously [49] but without phosphatase treatment. Proteins were re-suspended and incubated in 100 mM Tris–HCl pH 8.0, 1% SDS, 1 mM EDTA, 1 mg/ml PMSF containing 25 mM AMS or 25 mM NEM for 30 min at 25 °C then 5 min at 37 °C. *Human embryonic kidney cells*: A 10 cm plate of confluent HEK293 cells was washed three times with Dulbecco’s phosphate-buffered saline (DPBS) (Sigma), then incubated for 10 min at 37 °C in DPBS supplemented, as indicated, with H_2_O_2_ (Sigma). Cells were washed three times in DPBS and then re-suspended in 500 µl of lysis buffer (50 mM Tris–HCl pH 7.5, 150 mM NaCl, 0.5% NP40 (IGEPAL), 10 mM imidazole, 2 µg/ml pepstatin, 2 µg/ml leupeptin, 100 µg/ml PMSF and 1%(v/v) aprotinin). Western blotting: Insoluble material was pelleted by centrifugation (13,000 rpm, 3 min). The concentration of the solubilised proteins (supernatant) was determined using a Pierce® BCA protein assay kit (Thermo Scientific). Protein samples were mixed with an equal volume of 2× SDS loading dye (625 mM Tris–HCl, pH 6.7, 50%(v/v) glycerol, 10% sodium dodecyl sulfate (SDS), 0.5% Bromophenol Blue) and sample volumes equivalent to 2 µg of protein were analyzed by 15% SDS-PAGE and western blotting. Tpx1 was detected using anti-Tpx1 polyclonal antibodies [12] and hyperoxidized Prx were detected with monoclonal anti-peroxiredoxin-SO3 antibodies (LabFrontiers) [26]. FlagTrx1 was detected with mononclonal anti-Flag (M2-Sigma) antibodies. For *S. pombe* anti-tubulin antibodies and for HEK293 cells anti-actin (Sigma) antibodies were used to confirm that gels were evenly loaded. Primary antibodies were diluted 1 in 1000 in TBST (1 mM Tris–HCl pH 8.0, 15 mM NaCl, 0.01%(v/v) Tween 20). As appropriate, HRP-conjugated anti-rabbit or anti-mouse IgG secondary antibodies (Sigma) were used, followed by the fluorescent substrate ECL Plus to visualize antibody-labeled proteins (Thermo Scientific). Digital images of western blots were acquired with a Typhoon™ 9400 (GE Healthcare) and densitometry analysis performed using ImageQuantTL (Version 7).

### 4.3 Hydrogen peroxide colorimetric quantitation in media

PeroXOquant Quantitative Peroxide Assay kit with aqueous compatible formulation (Thermo scientific) was used according to manufacturer’s protocol. Briefly, *S. pombe* cells were grown in EMM media to OD_600_ 0.5. H_2_O_2_ was added to the growing cultures at a final concentration of 50 μM. 20 μl of media taken at various time points as indicated, were mixed in a 96-well microplate with 200 μl of working solution (freshly prepared according to manufacturer’s instructions). The mix was incubated at room temperature for 20 min. Absorbance at 560 nm was measured using a TECAN infinite M200 plate reader. The blank value (EMM without H_2_O_2_) was automatically subtracted from all sample measurements.

### 4.4 Measuring changes in intracellular H_2_O_2_ concentration

The H_2_O_2_ sensor, acetylated Peroxyfluorescein 3 (PF3) [25], was added to 10 ml (2×10^7^) exponentially growing *S. pombe* cells (OD_595_ 0.4–0.5) to a final concentration of 5 µM. Cells were incubated in the dark for 20 min, washed once then re-suspended in an equal volume of PBS (137 mM NaCl, 2.7 mM KCl, 10 mM Na_2_HPO_4_, and 1.8 mM KH_2_PO_4_ pH 7.4). HEK293 cells were incubated for 20 min in DPBS containing 5 µM PF3, washed three times then re-suspended in DPBS to a final concentration of 2×10^6^ cells/ml. Following re-suspension, 200 µl aliquots (4×10^6^ *S. pombe* cells, 4×10^5^ HEK293 cells) of PF3-labeled cells, unlabeled cells or PBS controls were transferred to a 96-well plate. Fluorescence measurements were made at 529 nm following excitation at 495 nm using a TECAN Infinite M200PRO plate reader and the average fluorescence of the unlabeled cells was deducted to calculate fluorescence due to PF3 (F). Measurements were taken 0 and 60 s before addition of H_2_O_2_ to determine the basal rate of reaction of PF3 with endogenously produced H_2_O_2_

(1) $${\left(\frac{\Delta F}{\Delta t}\right)}_{\mathrm{no}\_\mathrm{stress}}=\frac{F_{t60}-F_{t0}}{60}$$

Fluorescence measurements were made 30, 60 and 120 s following addition of H_2_O_2_ (0–500 µM) and used to calculate the rate of change of fluorescence.

(2) $${\left(\frac{∆F}{∆t}\right)}_{\mathrm{stress}}=\frac{F_{t120}-F_{t30}}{90}$$

The rate of fluorescence change due to exogenous H_2_O_2_ (∆*F*/∆*t*) was then calculated using Eq. (3) and plotted against H_2_O_2_ concentration using the graphics and statistics package R.

(3) $$\left(\frac{∆F}{∆t}\right){=\left(\frac{∆F}{∆t}\right)}_{\mathrm{stress}}-{\left(\frac{∆F}{∆t}\right)}_{\mathrm{no}\_\mathrm{stress}}$$

The R linear model function [50] was used to calculate the derivative and 95% confidence interval for the change (∆*F*/∆*t*) when H_2_O_2_ is increased from 0 to 100 µM H_2_O_2_ (*S. pombe*) or increased from 0 to 20 µM (HEK293 cells). This gradient was extrapolated using the equation *y*=*mx*+*c* and plotted on the same axis as the (∆*F*/∆*t*) data.

### 4.5 Computational method: Selection of a suitable reaction network for the kinetic model of Tpx1 oxidation

To find a set of reactions able to describe the formation of the Tpx1 monomers and disulfide homodimers detected in our experiments, we built a series of alternative models which contained different sets of biochemically feasible reactions. Parameter estimation was performed for each preliminary model, including preliminary models A and B (Fig. 5 and Table S4) (see below for details), and the model with parameters similar to published values that gave the lowest Akaike information criterion (AIC) parameter set [51] was selected for the final model (Fig. 3 and Table S5).

### 4.6 Computational Methods: Rate laws and measured parameters

The model contained two compartments, an extracellular compartment with volume *Vol*_*ex*_ (l) representing the growth media around the cells and an intracellular compartment of volume *Vol*_*int*_ (l) representing the total volume of all of the cells. The volume of the intracellular compartment was estimated using

(4) $${\mathrm{Vol}}_{\mathrm{int}}={\mathrm{Cell}}_{\mathrm{vol}}\times {\mathrm{Cell}}_{\mathrm{Num}}$$

where *Cell*_*Num*_=4×10^8^ (the number of cells in 50 ml of an OD_595_ 0.4 culture) and *Cell*_*Vol*_ is the mean volume of an *S. pombe* cell, measured for exponentially growing wild-type (972) cells (CASY®, Schärfe System) as 126 µM^3^. The rate of movement of H_2_O_2_ between these compartments was modeled to move down its concentration gradient using the rate equations Eq. (5) (H_2_O_2__influx) and Eq. (6) (H_2_O_2__efflux).

(5) $$v_{\mathrm{in}}=k_{H2O2\_\mathrm{perm}}\times {\left[H_{2}O_{2}\right]}_{\mathrm{ex}}$$

(6) $$v_{\mathrm{eff}}=k_{H2O2\_\mathrm{perm}}\times {\left[H_{2}O_{2}\right]}_{\mathrm{int}}$$

where $v_{\mathrm{in}}$ is the rate of influx and $v_{\mathrm{eff}}$=rate of efflux, [H_2_O_2_]_ex_ and [H_2_O_2_]_int_ represent the extracellular and intracellular H_2_O_2_ concentration and *k*_*H2O2_perm*_ is a constant representing all other factors that influence the rate of H_2_O_2_ movement between each compartment. The non-Tpx1 metabolism of H_2_O_2_ was modeled using Michaelis–Menten kinetics and all other reactions in the model were governed by mass action kinetics.

The parameters for the model were either derived from published work, measured experimentally or estimated from our experimental data. The initial concentrations of Tpx1 and Trx1 in the model were based on the global quantification of the *S. pombe* proteome [52]. The copy number per cell for these two proteins was used to calculate the concentration of each protein in the intracellular compartment using:

(7) $$M=\frac{\mathrm{cpc}}{N_{A}\times {\mathrm{Cell}}_{\mathrm{Vol}}}$$

where *M* is the molar protein concentration, *cpc*=copy number per cell [52] and *N*_*A*_ =Avagadro constant 6.02×10^23^ mol^−1^.

Rate constants for the oxidation of the peroxidatic cysteine (Cys_P_-SH) and the hyperoxidation of the sulfenic acid intermediate (Cys_P_-SOH) were taken from a recent study of human Prx1 and Prx3 [16]. Michaelis–Menten parameters for the reduction of Trx1 by Trr1 were based on those experimentally determined for the orthologous *S. cerevisiae* enzymes [53]. All other parameters were estimated from our experimental data using parameter estimation.

### 4.7 Computational Methods: Parameter estimation, time course simulation, identifiability analysis and data representation

Parameter estimation was performed in COPASI 4.13 [54]. The data set used for the parameter estimation was calculated from the relative intensities of the Tpx1 monomer and disulfide homodimer bands detected in 2–5 independent biological replicates of Tpx1 oxidation experiments depicted in Fig. 1A and Fig. 2A, B (Table S1), and PeroXOquant measurements for the removal of extracellular H_2_O_2_ (Fig. 3B and Table S1). Based on a broad range of experimental evidence (reviewed in [55]), a mock data set assuming a steady-state intracellular H_2_O_2_ concentration of 1 nM in exponentially growing cells was also included in the parameter estimation.

Parameter estimation was performed 500 times from random initial parameter values using the Levenberg–Marquardt algorithm [56] for each model (Table S2). The parameter set used for the final model had an AIC of 77.6 and was found on 327 out of 500 estimations, each of these 327 parameter estimations converged on similar values as indicated by the frequency distributions for each parameter (Fig. S3). One-dimensional likelihood profiles for each parameter and 95% confidence intervals were calculated using a simple identifiability analysis [57]. This analysis demonstrated that the estimated parameters were identifiable (Fig. S4) with acceptable 95% percent confidence regions calculated for each parameter (Table S3 and Fig. S4). Time course simulation was performed in COPASI 4.10 [54] using the deterministic (LSODA) algorithm. All graphics and further analysis of the simulation and identifiability data were performed using R.

## Conflict of interest

The authors declare that they have no conflict of interest.

## Author contributions

L.T. designed and performed experiments under the supervision of A.D. and E.V., L.T. developed and tested mathematical models under the supervision of D.S. and E.V. with input from G.S. and P.D.P., Z.U. contributed data to Figs. 7,8 and S5 under the supervision of E.V. and L.T. W.P. assisted L.T. with the generation of data in Fig. 6D–F with advice from B.D. and E.V. Experiment in Fig. 3B was carried out by C.R. under the supervision of J.B., B.D., T.B. and C.C. synthesized PF3 and advised on its use in experiments in Figs. 6–8. L.T. and E.V. wrote the manuscript with input from all authors.

## Footnotes

Appendix A Supplementary data associated with this article can be found in the online version at doi:10.1016/j.freeradbiomed.2016.02.035.
