# Supplementary material for: Increasing extracellular H2O2 produces a bi-phasic response in intracellular H2O2, with peroxiredoxin hyperoxidation only triggered once the cellular H2O2-buffering capacity is overwhelmed
Source: Free Radic Biol Med. 2016 Jun;95:333–48. doi: 10.1016/j.freeradbiomed.2016.02.035 (PMC4891068; doi:10.1016/j.freeradbiomed.2016.02.035)
Supplement: Supplementary file 1 — Supplementary material [file mmc1.pdf]

Table S1 Data used in parameter estimation and shown graphically in Figs.3- 5.
All times are in seconds. All concentrations are in µM. Concentrations of each Tpx1 form were based on the relative abundance of each form determined by analysis of western blots (Figs.1-2) and the cellular concentration of Tpx1, as calculated from the copies per cell and the cell volume (see experimental procedures for details).

Figs. 1, 3 and 5: Tpx1 undergoes oxidation to multiple redox states following exposure to different concentrations of H_2O_2:

Tpx1 oxidation states 20 seconds after 0 - 6000 µM H_2O_2 (exp1)

Time	H_2O_2	Tpx1SH	Tpx1SOOH	Tpx1ox#2	Tpx1ox#1	Tpx1ox:SOOH
0	0	4	0	0	0	0
20	0	3.04	0	0	0.48	0
20	50	1.3292	0	0.2036	1.1318	0
20	100	0.0708	0	1.0862	0.8784	0
20	200	0	0	1.318	0.4388	0.2434
20	300	0	0	1.2006	0.3672	0.4322
20	500	0	0	0.9796	0.3188	0.7018
20	1000	0	0	0.9254	0.2482	0.8264

Tpx1 oxidation states 20 seconds after 0 - 6000 µM H_2O_2 (exp2)

Time	H_2O_2	Tpx1SH	Tpx1SOOH	Tpx1ox#2	Tpx1ox#1	Tpx1ox:SOOH
0	0	4	0	0	0	0
20	0	3.71	0	0	0.145	0
20	50	1.35	0	0.175	1.155	0
20	100	1.26	0	0.165	1.2	0
20	200	0	0	0.965	0.56	0.47
20	300	0	0	1.04	0.485	0.475
20	500	0	0.04	0.955	0.31	0.715
20	1000	0	0.14	1.225	0	0.705

Figs. 2, 4 and 5: Changes in Tpx1 oxidation over time following treatment with 100 or 200µM H_2O_2:

100 µM H_2O_2 Time course data (exp1)

Time	H_2O_2	Tpx1SH	Tpx1SOOH	Tpx1ox#2	Tpx1ox#1	Tpx1ox:SOOH
0	100	4	0	0	0	0
30	100	2.439	0	0.385	0.396	0
60	100	2.528	0	0.365	0.371	0
120	100	2.396	0	0.379	0.422	0
300	100	1.578	0	0.495	0.716	0
600	100	1.746	0	0.525	0.602	0
0	0	4	0	0	0	0
20	0	3.95	0	0	0.025	0
60	0	3.95	0	0	0.025	0

100 µM H_2O_2 Time course data (exp2)

Time	H_2O_2	Tpx1SH	Tpx1SOOH	Tpx1ox#2	Tpx1ox#1	Tpx1ox:SOOH
0	100	4	0	0	0	0
20	100	0.978	0	0.523	0.988	0
40	100	1.002	0	0.475	1.024	0
60	100	1.144	0	0.466	0.961	0
0	0	4	0	0	0	0
20	0	3.3184	0	0	0.3408	0
60	0	3.3184	0	0	0.3408	0

100 µM H_2O_2 Time course data (exp3)

Time	H_2O_2	Tpx1SH	Tpx1SOOH	Tpx1ox#2	Tpx1ox#1	Tpx1ox:SOOH
0	100	4	0	0	0	0
60	100	0.999	0	0.598	0.903	0
300	100	1.13	0	0.43	1.005	0
600	100	2.07	0	0.25	0.715	0
0	0	4	0	0	0	0
20	0	3.4872	0	0	0.5128	0
60	0	3.4872	0	0	0.5128	0

100 µM H_2O_2 Time course data (exp4)

Time	H_2O_2	Tpx1SH	Tpx1SOOH	Tpx1ox#2	Tpx1ox#1	Tpx1ox:SOOH
0	100	4	0	0	0	0
60	100	0.127	0	0.64	1.297	0
300	100	0.423	0	0.393	1.396	0
600	100	0.85	0	0.306	1.269	0
0	0	4	0	0	0	0
20	0	3.1964	0	0.0616	0.4018	0
60	0	3.1964	0	0.0616	0.4018	0

200 µM H_2O_2 Time course data (exp1)

Time	H_2O_2	Tpx1SH	Tpx1SOOH	Tpx1ox#2	Tpx1ox#1	Tpx1ox:SOOH
0	200	4	0	0	0	0
20	200	0.07	0	0.91	0.77	0.28
40	200	0.09	0	0.81	0.84	0.31
60	200	0.03	0	0.9	0.83	0.26
120	200	0	0.43	0.76	0.71	0.32
240	200	0	1.69	0.44	0.52	0.2
360	200	0	0.82	0.5	0.77	0.33
480	200	0	1.2	0.41	0.68	0.31
600	200	0	0.95	0.43	0.75	0.35
0	0	4	0	0	0	0
20	0	3.09	0	0	0.227	0
60	0	3.09	0	0	0.227	0

200 µM Tpx1 Time course data (exp2)

Time	H_2O_2	Tpx1SH	Tpx1SOOH	Tpx1ox#2	Tpx1ox#1	Tpx1ox:SOOH
0	200	4	0	0	0	0
20	200	0.185	0	0.72	0.838	0.349
40	200	0.195	0	0.631	0.914	0.357
60	200	0.198	0	0.592	0.89	0.419
120	200	0	1.749	0.234	0.632	0.259
240	200	0	3.382	0.047	0.197	0.065
360	200	0	3.228	0.045	0.23	0.111
480	200	0	3.003	0.061	0.32	0.117
600	200	0	3.088	0.044	0.211	0.201
0	0	4	0	0	0	0
20	0	3.30	0	0	0.348	0
60	0	3.30	0	0	0.348	0

200 µM H_2O_2Time course data (exp3)

Time	H_2O_2	Tpx1SH	Tpx1SOOH	Tpx1ox2	Tpx1ox1	Tpx1oxSOOH
0	200	4	0	0	0	0
600	200	0	1.256	0.334	0.829	0.209
0	0	4	0	0	0	0
20	0	3.492	0	0	0.254	0
60	0	3.492	0	0	0.254	0

200 µM H_2O_2Time course data (exp4)

Time	H_2O_2	Tpx1SH	Tpx1SOOH	Tpx1ox#2	Tpx1ox#1	Tpx1ox:SOOH
0	200	4	0	0	0	0
20	200	0.0592	0	0.8176	0.8848	0.2682
40	200	0.0736	0	0.8752	0.8784	0.2096
60	200	0	0	0.8938	0.9174	0.1888
120	200	0	0.2072	0.8656	0.755	0.2758
240	200	0	0.8752	0.5096	0.8218	0.2308
360	200	0	1.1464	0.4876	0.7414	0.1978
480	200	0	1.6988	0.3452	0.654	0.1516
600	200	0	1.8436	0.2448	0.6148	0.2186
0	0	4	0	0	0	0
20	0	3.2296	0	0	0.3852	0
60	0	3.2296	0	0	0.3852	0

200 µM Tpx1 Time course data (exp5)

Time	H_2O_2	Tpx1SH	Tpx1SOOH	Tpx1ox#2	Tpx1ox#1	Tpx1ox:SOOH
0	200	4	0	0	0	0
30	200	0.0372	0.1324	0.7852	0.8534	0.2766
60	200	0	0.712	0.5828	0.8456	0.2154
120	200	0	1.7392	0.3964	0.5908	0.1434
240	200	0	3.1276	0.2348	0.2014	0
360	200	0	2.7188	0.2132	0.4276	0

480	200	0	2.9104	0.204	0.3408	0
600	200	0	2.8304	0.1632	0.4216	0
0	0	4	0	0	0	0
20	0	3.366	0	0.2188	0.4152	0
60	0	3.366	0	0.2188	0.4152	0

Fig. 3B: Change in extracellular H_2O_2 concentration with time following addition of 50μM H_2O_2

Exp1:

Time	H_2O_2ex_Exp1	H_2O_2initial
0	50	50
60	48	50
120	48	50
300	43	50
600	40	50
900	38	50
1200	34	50
1500	29	50
1800	24	50

Exp2:

Time	H_2O_2ex_Exp2	H_2O_2initial
0	50	50
60	49	50
120	49	50
300	47	50
600	42	50
900	38	50
1200	34	50
1500	30	50
1800	27	50

Intracellular H_2O_2 data:

Time	H_2O_2ex	H_2O_2int
0	0	0.001
10	0	0.001
60	0	0.001

Table S2 (Related to Fig. 3). Rate laws for each of the reactions included in the Prx oxidation model depicted in Fig. 3

Reaction	Rate law
H_2O_2_influx	k_{H2O2_perm} $[H_2O_{2ex}]$
H_2O_2_efflux	k_{H2O2_perm} $[H_2O_{2int}]$
H_2O_2_metab	$V_{max\_H2O2\_metab}[H_2O_{2int}]/K_{m\_H2O2\_metab} + [H_2O_{2int}]$
H_2O_2_basal	V_{basal}
Cys_P_ox1	k_{cys_ox}[Tpx1SH]$[H_2O_{2int}]$
disulph_form1a	$k_{disulph\_form1}$[Tpx1SOH][Tpx1SH]
disulph_red1	$k_{disulph\_red1}$[Trx1red][Tpx1ox#1]
Cys_P_ox2	k_{cys_ox}[Tpx1SH]$[H_2O_{2int}]$
disulph_form1b	$k_{disulph\_form1}$[Tpx1SOH][Tpx1SOH]
disulph_form2	$k_{disulph\_form2}$[Tpx1ox:SOH]
disulph_red2	$k_{disulph\_red2}$[Trx1red][Tpx1ox#2]
hyp_ox	k_{hyp_ox}[Tpx1ox:SOH]$[H_2O_{2int}]$
disulph_red3	$k_{disulph\_red3}$[Trx1red][Tpx1ox:SOOH]
Trx1_reduction	k_{Trx_red}[Trx1-ox]

Table S3 (Related to Fig. 3). Parameters used in the final model of Prx oxidation.

Parameter	Value	Units	Source	95% Confidence Interval
[Tpx1SH]	4	µM	Marguerat et al, (2012)	NA
[Trx1red]	0.7	µM	Marguerat et al, (2012)	NA
k_{H2O2_perm}	1.72 x 10^{-5}	l s^{-1}	Parameter Estimation	1.65 x 10^{-5} – 1.8 x 10^{-5}
$V_{max\_H2O2\_metab}$	59	µM s^{-1}	Parameter Estimation	51 - 70
$K_{m\_H2O2\_metab}$	0.007	µM	Parameter Estimation	0.003 - 0.053
k_{cys_ox}	20	$µM^{-1}$ s^{-1}	Peskin et al, (2013)	NA
$k_{disulph\_form1}$	1.01	$µM^{-1}$ s^{-1}	Parameter Estimation	0.25 - 10.97
$k_{disulph\_form2}$	3.44	s^{-1}	Parameter Estimation	1.47 - 42.93
$k_{disulph\_red1}$	0.190	$µM^{-1}$ s^{-1}	Parameter Estimation	0.087 - 1.538
$k_{disulph\_red2}$	0.143	$µM^{-1}$ s^{-1}	Parameter Estimation	0.058 - 1.312
$k_{disulph\_red3}$	0.029	$µM^{-1}$ s^{-1}	Parameter Estimation	0.017 - 0.048
k_{Trx_red}	34	$µM^{-1}$ s^{-1}	Oliveira et al, (2010)	NA
k_{hyp_ox}	0.012	$µM^{-1}$ s^{-1}	Peskin et al, (2013)	NA
Vol_{ex}	0.05	l	Measured	NA
Vol_{int}	5.20E-05	l	Measured	NA
V_{basal}	5.28	µM s^{-1}	Parameter Estimation	1.48 - 16.20

Table S4 (Related to Fig. 5) Parameter sets used in Alternative Models A and B.

Parameter	Alternative Model A	Alternative Model B	Source
**[Tpx1SH]**	4 µM	4 µM	Marguerat et al, 2012
**[Trx1red]**	0.7 µM	0.7 µM	Marguerat et al, 2012
k_{H2O2_perm}	1.72 x 10^{-5} l s^{-1}	1.41 x 10^{-5} l s^{-1}	Parameter Estimation
$V_{max\_H2O2\_metab}$	376 µM s^{-1}	NA	Parameter Estimation
$K_{m\_H2O2\_metab}$	0.133 µM	NA	Parameter Estimation
k_{cys_ox}	20 $µM^{-1}$ s^{-1}	20 $µM^{-1}$ s^{-1}	Peskin et al, 2013
k_{cys_ox3}*	0.016 $µM^{-1}$ s^{-1}	NA	Parameter Estimation
$k_{disulph\_form1}$	807.00 $µM^{-1}$ s^{-1}	1200.00 $µM^{-1}$ s^{-1}	Parameter Estimation
$k_{disulph\_form2}$	4.44 s^{-1}	500.00 s^{-1}	Parameter Estimation
$k_{disulph\_red1}$	4.815 $µM^{-1}$ s^{-1}	91.805 $µM^{-1}$ s^{-1}	Parameter Estimation
$k_{disulph\_red2}$	0.404 $µM^{-1}$ s^{-1}	100.000 $µM^{-1}$ s^{-1}	Parameter Estimation
$k_{disulph\_red3}$	0.024 $µM^{-1}$ s^{-1}	0.005 $µM^{-1}$ s^{-1}	Parameter Estimation
k_{Trx_red}	34 $µM^{-1}$ s^{-1}	34 $µM^{-1}$ s^{-1}	Oliveira et al, 2010
k_{hyp_ox}	0.012 $µM^{-1}$ s^{-1}	0.012 $µM^{-1}$ s^{-1}	Peskin et al, 2013
Vol_{ex}	0.05l	0.05l	Measured
Vol_{int}	5.20E-05l	5.20E-05l	Measured
V_{basal}	45.53 µM s^{-1}	0.01 µM s^{-1}	Parameter Estimation

* k_{cys_ox3} refers to the reaction of Tpx1ox#1 with H_2O_2 to form Tpx1ox:SOH which has the rate law k_{cys_ox3}[Tpx1ox#1][H_2O_2int].

Table S5 (Related to Fig. 3-5). Parameter Estimation Results Summary for Final Model and for Alternative Models A and B

	Final Model	Alternative Model A	Alternative Model B
**Parameter sets with unique AIC/SSR**	141	146	137
**Lowest SSR para set**	128	132	509
**Lowest AIC set**	78	88	75
**Times lowest AIC/SSR was found (in 500)**	327	218	1

Table S6 (Related to Figs. 8 and S5). Rate laws for Thiol-proteome model

Reaction	Rate law
H_2O_2_influx	$k_{H2O2\_perm} \cdot [H_2O_{2ex}]$
H_2O_2_efflux	$k_{H2O2\_perm} \cdot [H_2O_{2int}]$
H_2O_2_basal	V_{basal}
Pr-SH oxidation	$k_{Pr\text{-}SH\_ox}[Pr\text{-}SH][H_2O_{2int}]$
Pr-SH reduction (Trx1)	$k_{Pr\text{-}SS\_red\_Trx1}[Pr\text{-}SS][Trx1red]$
Pr-SH reduction (Txl1)	$k_{Pr\text{-}SS\_red\_Txl1}[Pr\text{-}SS][Txl1red]$
Trx1 reduction by Trr1	$k_{Trx\_red}[Trr1][Trx1ox]$
Txl1 reduction by Trr1	$k_{Txl1\_red}[Trr1][Txl1ox]$

Table S7 (related to Figs. 8 and S5). Parameters for Thiol-proteome model

Parameter	Value	Units	Source
[Trx1red]	0.7	µM	Marguerat et al, (2012)
[Trx1ox]	0	µM	Assigned
[Txl1red]	0.17	µM	Marguerat et al, (2012)
[Txl1ox]	0	µM	Assigned
[Trr1]	1	µM	Marguerat et al, (2012)
[Pr-SH]	13,000	µM	Hansen et al, (2009)
[Pr-SS]	0	µM	Assigned
k_{H2O2_perm}	1.72×10^{-5}	$l\ s^{-1}$	Parameter Estimation*
$k_{Pr\text{-}SH\_ox}$	0.0005	$\mu M^{-1}\ s^{-1}$	Assigned
$k_{Pr\text{-}SS\_red\_Trx1}$	0.001^{Δ}	$\mu M^{-1}\ s^{-1}$	Assigned
$k_{Pr\text{-}SS\_red\_Txl1}$	0.01	$\mu M^{-1}\ s^{-1}$	Assigned
k_{Trx_red}	33.6	$\mu M^{-1}\ s^{-1}$	Oliveira et al, (2010)
k_{Txl1_red}	33.6	$\mu M^{-1}\ s^{-1}$	Assigned[†]
Vol_{ex}	0.05	l	Measured*
Vol_{int}	5.20E-05	l	Measured*
V_{basal}	1	$\mu M\ s^{-1}$	Assigned

*These parameters were chosen to be the same as in the Tpx1 oxidation model (Table S3).

[†]The rate constant for the reduction of Txl1 by Trr1 (k_{Txl1_red}) was assumed to be the same as k_{Trx_red}.

[Δ] The parameter $k_{Pr\text{-}SS\_red\_Trx1}$ was assigned as 1 $\mu M^{-1}\ s^{-1}$ to generate Fig. S5A.

Table S8 (Related to Experimental Procedures). ***S. pombe*** **strains used in this study.**

Strain	Genotype	Source
**972**	*h^-*	Lab stock
**NT4**	*h^+ ade6-M216 leu1-32 ura4-D18*	Lab stock
**CHP429**	*h^- ade6-M216 leu1-32 his7-366 ura4-D18*	Lab stock
**LT3**	*h^+ade6-M216 leu1-32 ura4-D18 ctt1::kan^{mx4}*	Dr. Peter Banks
**JR20**	*h^+ade6-M210 leu1-32 ura4-D18 tpx1::$ura4^+$ $tpx1^{1\text{-}181}$:LEU2*	Day *et al.*, 2012
**JR68**	*h^+ ade6 leu1-32 ura4-D18 tpx1::$ura4^+$ $tpx1^+$:LEU2*	Day *et al.*, 2012
**AD140**	*h^+ ade6 his7-366 leu1-32 ura4-D18 txl1::kan^{mx4} trx1::kan^{mx4}*	Brown *et al.*, 2013
**JB35**	*h^- ade6-M216 leu1-32 ura4-D18 his7-366 trx1::kan^{mx4} Flag-trx1:ura4*	Day *et al.*, 2012
**SB13**	*h^+ade6 leu1-32 ura4-D18 gpx1::ura4 his7-366*	Bozonet *et al.*, 2005
**VX00**	*h^+ade6 leu1-32 ura4-D18 tpx1::ura4 his7-366*	Veal *et al.*, 2004

Figure S1 (Related to Fig. 1) Analysis of Tpx1-containing complexes [A] confirming that they represent disulfides and [B] identifying that the 55kD band represents Trx1-Tpx-1-Tpx1 disulfides The sensitivity of Tpx1-containing bands with MW>20kD to reduction by beta-mercaptoethanol to a 20kD band indicates that they represent disulfide-bonded forms of Tpx1. Western blot analysis, using anti-Tpx1 antibodies, of wild-type (972) cells treated for 20s with 0–1000µM H_2O_2. *In vivo* patterns of thiol oxidation were preserved using NEM and samples were treated, as indicated, with beta-mercaptoethanol(βME) prior to electrophoretic separation. **[B]** The decreased mobility of the 55kD complex in cells expressing FlagTrx1 instead of Trx1 is consistent with the increase in MW expected for a complex representing a Tpx1-Tpx1-FlagTrx1 rather than a Tpx1-Tpx1-Trx1 disulfide. Western blot analysis of Tpx1 in wild-type cells expressing wild-type Trx1 (CHP429) or Flag epitope-tagged Trx1 (JB35) treated for 30s with 1mM H_2O_2 and analysed with anti-Tpx1 antibodies.

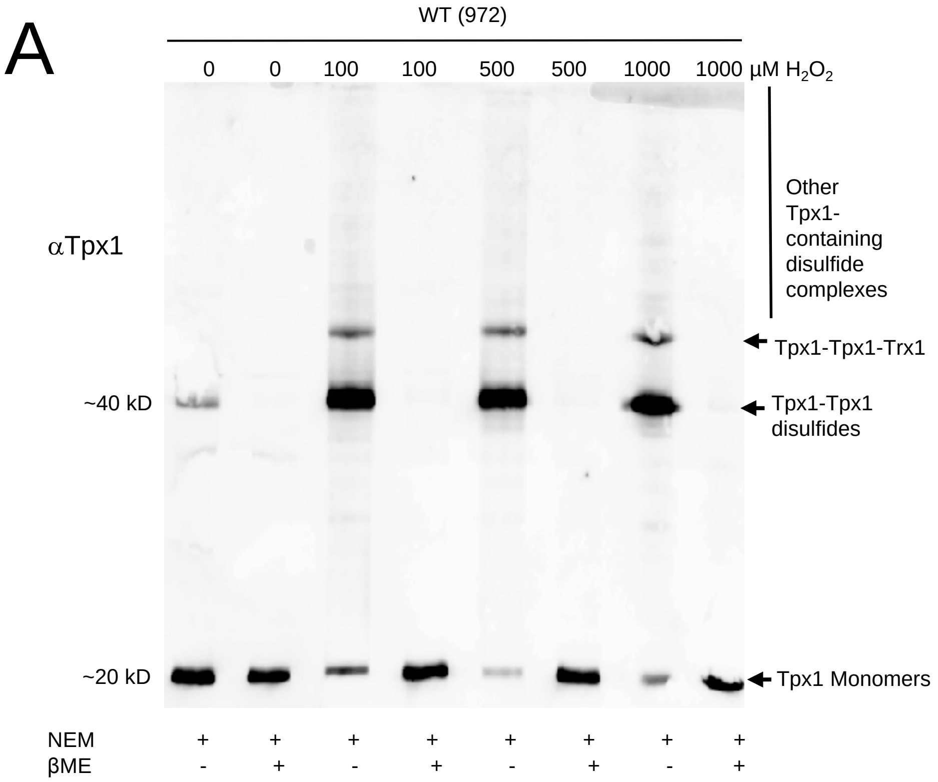

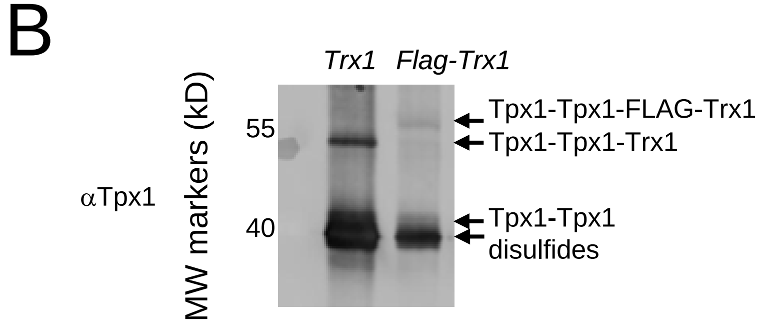

Figure S2 (Related to Fig.2). The kinetics of formation of hyperoxidized Tpx1 following exposure to increasing concentrations of H_2O_2 Western blot analysis of levels of hyperoxidized Tpx1 in wild-type (972) *S. pombe* cells before and following exposure, for the indicated time, to 100, 200, 500 or 1000µM H_2O_2. Hyperoxidized Tpx1 (Tpx1SOOH and Tpx1ox:SOOH) was detected using antibodies specific to forms of Prx in which the CysP is sulfinylated or sulfonylated (αPrxSO3).

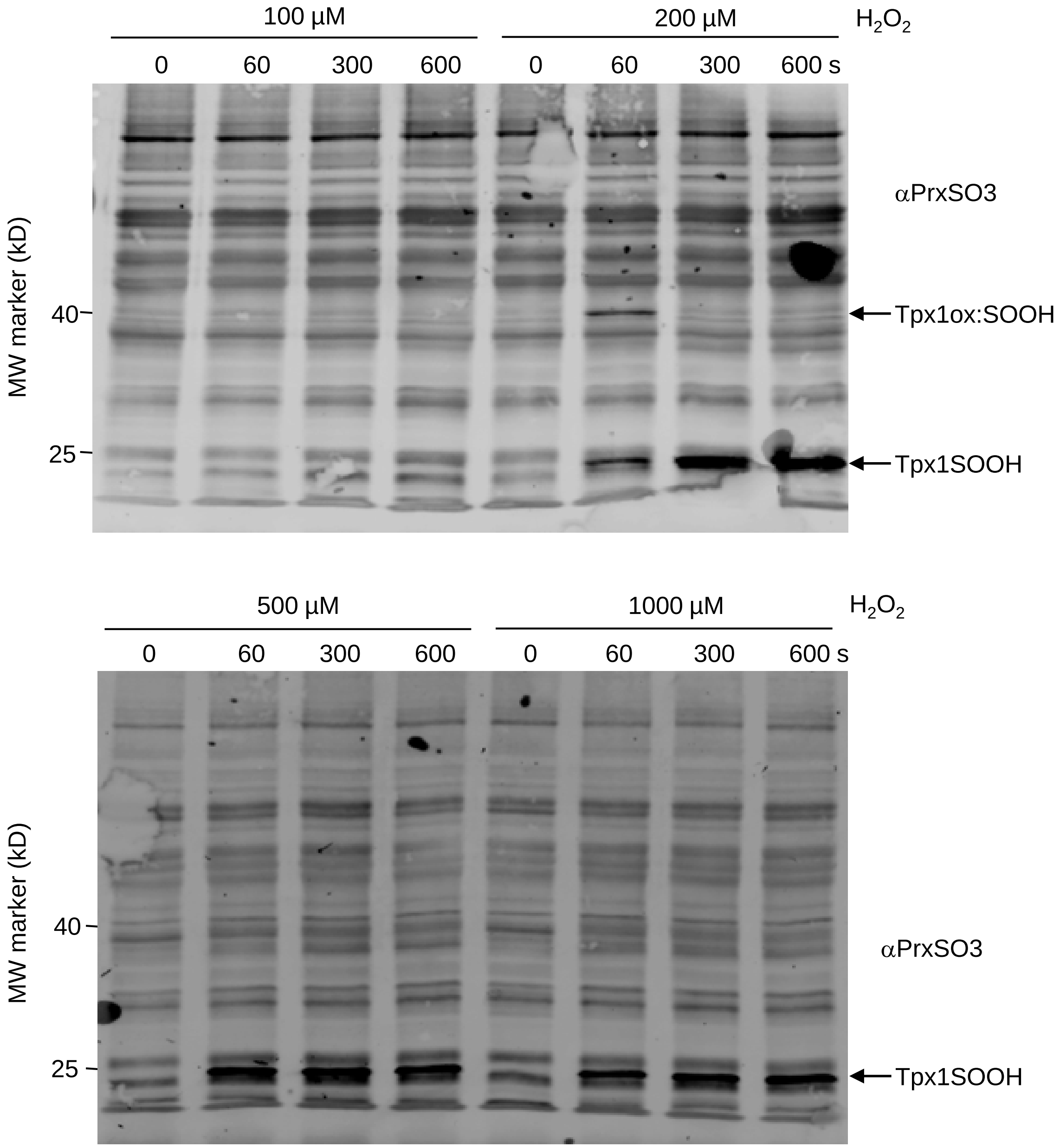

Figure S3 (Related to Fig. 3) Frequency distributions for the parameter sets estimated for the final model of Tpx1 oxidation indicate good convergence for each parameter. Parameter estimation found 327 parameter sets with an AIC of 77.6. The frequency distributions for each parameter in these 327 parameter sets are shown. Each parameter displayed a normal distribution indicating that all 327 parameter sets were predicting a similar value for each parameter.

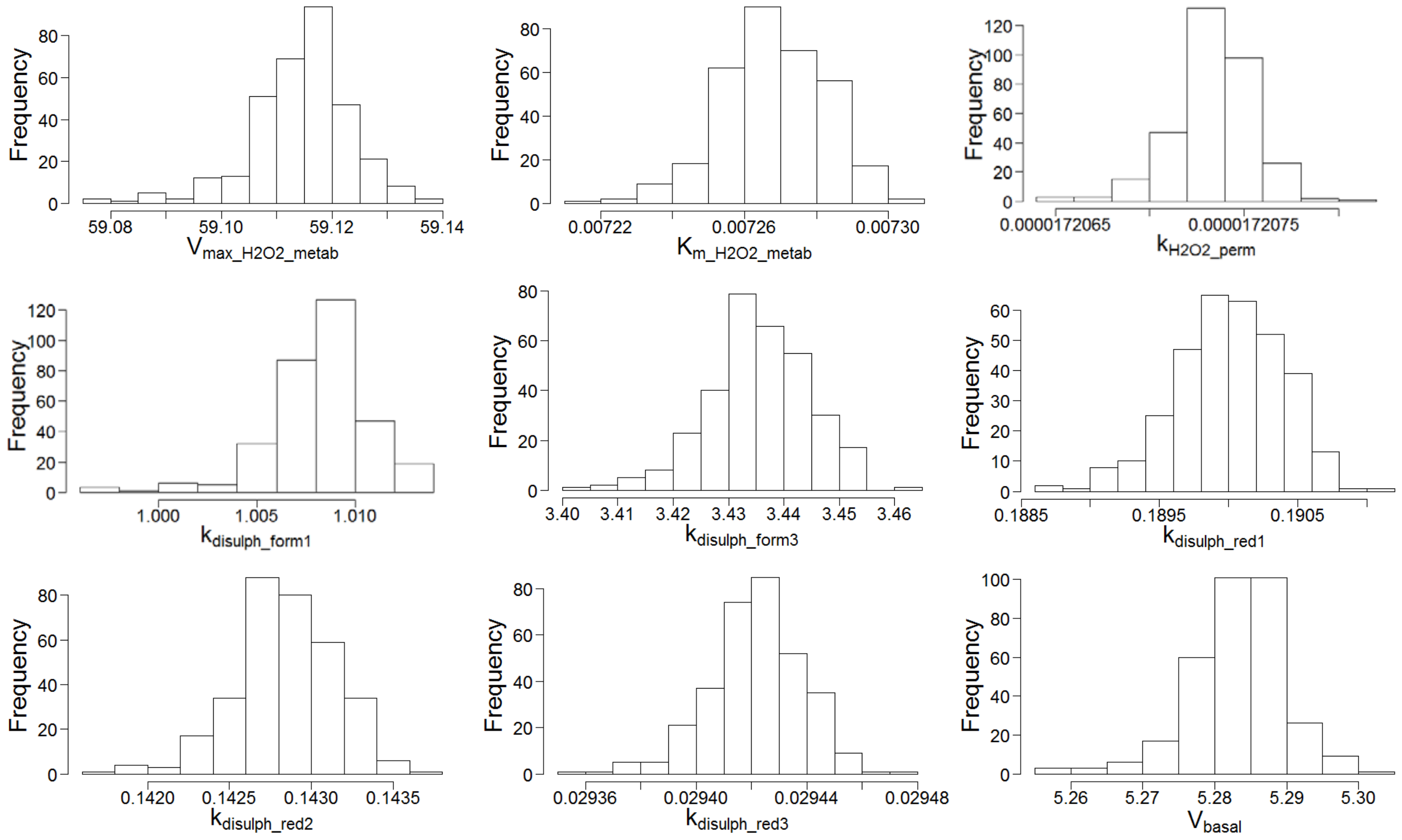

Figure S4 (Related to Fig. 3). Identifiability analysis for the parameters used in the final model of *in vivo* Tpx1 oxidation. One-dimensional contour plots for each of the parameters in the model calculated using identifiability analysis. The dotted lines represent the 50% and the dashed lines the 95% confidence intervals for each parameter, as indicated.

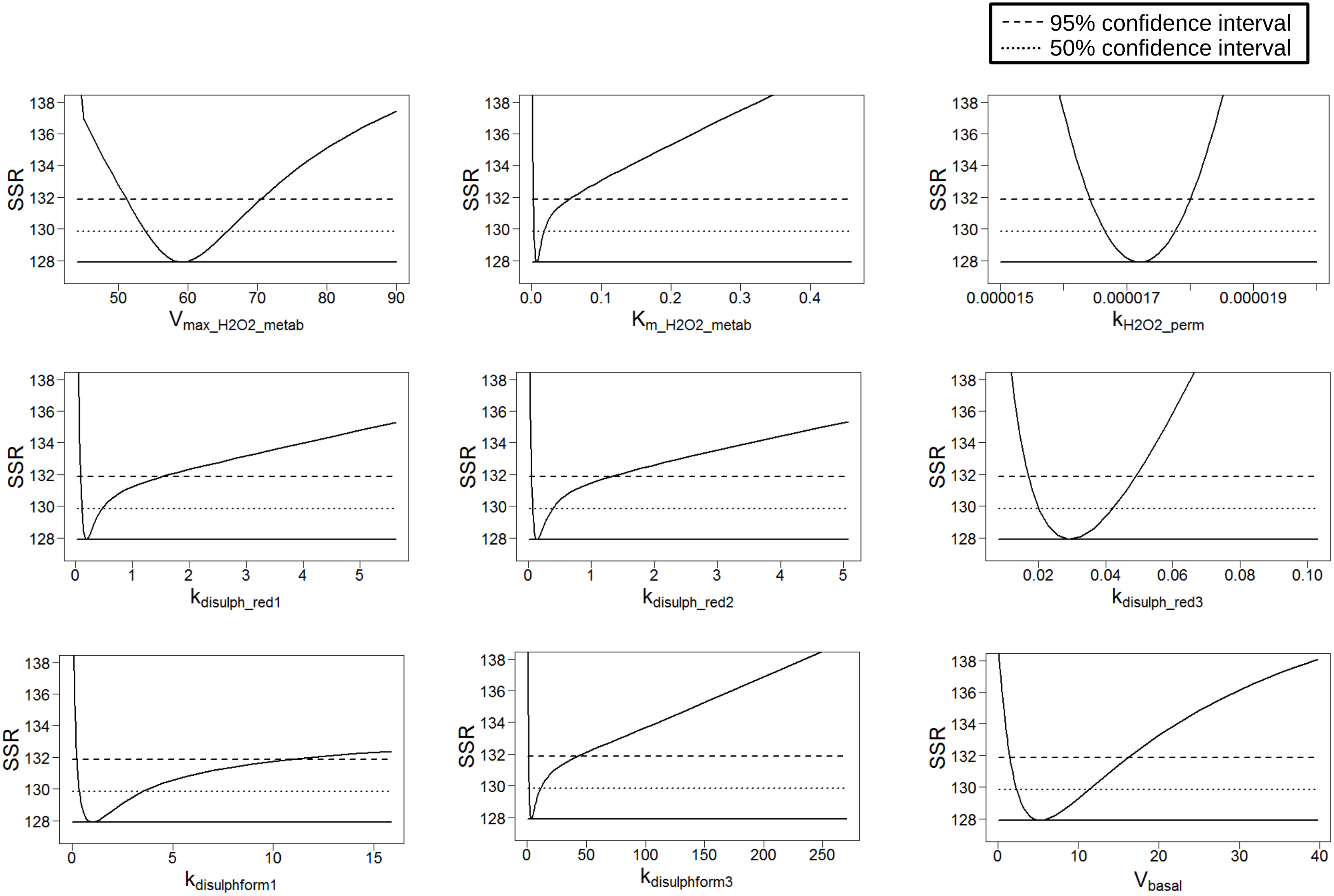

Figure S5 (related to Fig. 8). The model (Tables S6 and S7) predicts that [A] the reactivity of Trx1 with protein disulfides (Pr-SS) $k_{Pr\text{-}SS\_red\_Trx1}$ **[B] Protein thiol (Pr-SH) concentration, will affect the ability of the thiol proteome to prevent rises in intracellular H_2O_2 concentration in response to increasing extracellular H_2O_2 (compare with Fig. 8F).**

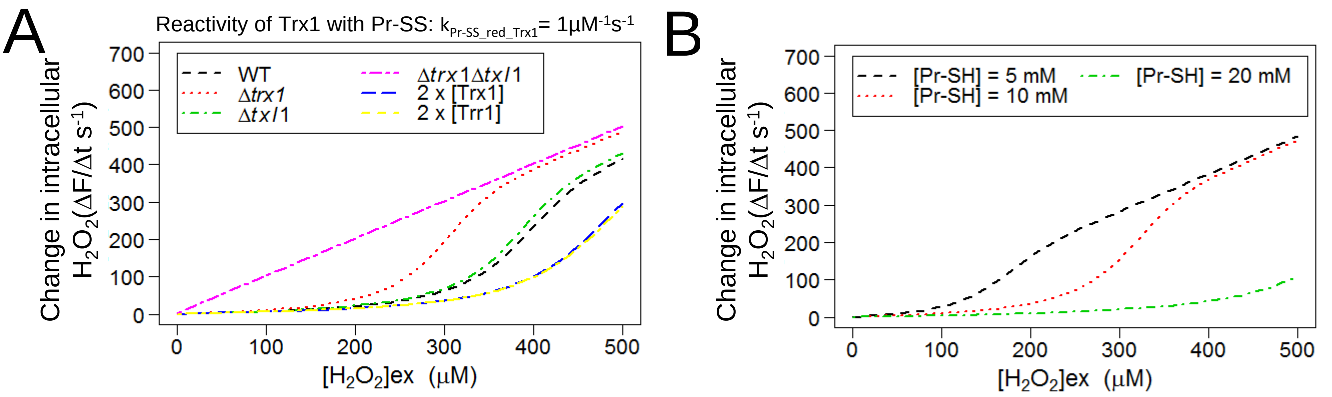

Supplemental References

Brown, J.D., Day, A.M., Taylor, S.R., Tomalin, L.E., Morgan, B.A., and Veal, E.A. (2013). A peroxiredoxin promotes H_2O_2 signaling and oxidative stress resistance by oxidizing a thioredoxin family protein. Cell Rep *5*, 1425-1435.

Day, A.M., Brown, J.D., Taylor, S.R., Rand, J.D., Morgan, B.A., and Veal, E.A. (2012). Inactivation of a peroxiredoxin by hydrogen peroxide is critical for thioredoxin-mediated repair of oxidized proteins and cell survival. Mol Cell *45*, 398-408.

Marguerat, S., Schmidt, A., Codlin, S., Chen, W., Aebersold, R., and Bahler, J. (2012). Quantitative analysis of fission yeast transcriptomes and proteomes in proliferating and quiescent cells. Cell *151*, 671-683.

Peskin, A.V., Dickerhof, N., Poynton, R.A., Paton, L.N., Pace, P.E., Hampton, M.B., and Winterbourn, C.C. (2013). Hyperoxidation of peroxiredoxins 2 and 3: rate constants for the reactions of the sulfenic acid of the peroxidatic cysteine. J Biol Chem *288*, 14170-14177.

Veal, E.A., Findlay, V.J., Day, A.M., Bozonet, S.M., Evans, J.M., Quinn, J., and Morgan, B.A. (2004). A 2-Cys peroxiredoxin regulates peroxide-induced oxidation and activation of a stress-activated MAP kinase. Mol Cell *15*, 129-139.
